# How is parental education associated with infant and young child feeding in Bangladesh? a systematic literature review

**DOI:** 10.1186/s12889-023-15173-1

**Published:** 2023-03-17

**Authors:** Plabon Sarkar, M. A. Rifat, Progati Bakshi, Imdadul Haque Talukdar, Sarah M. L. Pechtl, Tobias Lindström Battle, Sanjib Saha

**Affiliations:** 1 Caritas Bangladesh, 2, Outer Circular Road, Shantibagh, Dhaka, 1217 Bangladesh; 2 Department of Global Public Health, Karolinska Institutet, Stockholm, 17177 Sweden; 3 Department of Food and Agroprocess Engineering, Bangabandhu Sheikh Mujibur Rahman Science and Technology University, Gopalganj, 8100 Bangladesh; 4 Department of Learning, Informatics, Management and Ethics (LIME), Karolinska Institutet, Stockholm, Sweden; 5 Department of Clinical Sciences, Health Economics Unit, Lund University, 22381 Lund, Sweden

**Keywords:** Breastfeeding, Complementary feeding, Infant feeding, Child feeding, Exclusive breastfeeding, Parental education, Maternal education, Bangladesh, Lower middle-income countries

## Abstract

**Background:**

Education is expected to bring about positive behavioral changes which could lead to improved health behaviors. Parental education is a primary determinant of child health and development. However, some evidence showed inverse associations between high parental education and recommended infant and young child feeding (IYCF) in Bangladesh. How the association of parental education differs with specific IYCF components has not been reviewed. Therefore, the role of parental education on optimal IYCF practices in Bangladesh appears to be inconclusive. The objective of this review is to summarize how parental education is associated with IYCF practices in Bangladesh.

**Method:**

This review was conducted following the Preferred Reporting Items for Systematic Reviews and Meta-Analyses (PRISMA) guideline. A systematic literature search was conducted in PubMed, Web of Science, Embase, and Google Scholar. Record searching, study selection, and data extraction was performed using Endnote online and Covidence tool, respectively. The Newcastle–Ottawa scale was used for quality assessment of the included studies.

**Results:**

Out of 414 initial hits, 34 studies were included for this review. Of the included studies, 32 were cross-sectional, one was a randomized controlled trial, and one was a retrospective cohort. Most of the studies (*n* = 24) were nationally representative whereas 10 studies had populations from district and sub-district level. Included studies considered different IYCF-related indicators, including breastfeeding (*n* = 22), complementary feeding (*n* = 8), both breastfeeding and complementary feeding (*n* = 2), both breastfeeding and bottle feeding (*n* = 1), and pre-lacteal feeding (*n *= 1). Parental education was found to be positively associated with complementary feeding practices. However, the role of parental education on breastfeeding, in general, was ambiguous. High parental education was associated with bottle-feeding practices and no initiation of colostrum.

**Conclusion:**

Public health interventions need to focus not only on non- and/or low-educated parents regarding complementary feeding but also on educated mothers for initiation of colostrum and proper breastfeeding practices.

**Trial registration:**

This systematic review is registered to PROSPERO (https://www.crd.york.ac.uk/prospero/) with registration ID: CRD42022355465.

**Supplementary Information:**

The online version contains supplementary material available at 10.1186/s12889-023-15173-1.

## Introduction

Infant and young child feeding (IYCF) practices are associated with the development and nutritional status of children and, ultimately, impact their health in later life [1]. Globally, inappropriate IYCF practices lead to childhood undernutrition which causes approximately 2.7 million child deaths annually, representing 45% of all child deaths [2]. More than 823,000 under-five deaths could be prevented every year in 75 lower middle-income countries if all children below 23 months were optimally breastfed [3]. Therefore, IYCF is a key area for child survival and promoting healthy growth and development [2].

According to the latest recommendations by the World Health Organization (WHO), optimal IYCF practices consist of 17 indicators of which six are related to breastfeeding, nine are related to complementary feeding, and two are related to other aspects. These indicators are: 1) ever breastfed, 2) early initiation of breastfeeding, 3) exclusively breastfed for the first two days after birth, 4) exclusive breastfeeding under six months, 5) mixed milk feeding under six months, 6) continued breastfeeding 12–23 months, 7) introduction of solid, semi-solid, or soft foods 6–8 months, 8) minimum dietary diversity 6–23 months, 9) minimum meal frequency 6–23 months, 10) minimum milk feeding frequency for non-breastfed children 6–23 months, 11) minimum acceptable diet 6–23 months, 12) egg and/or flesh food consumption 6–23 months, 13) sweet beverage consumption 6–23 months, 14) unhealthy food consumption 6–23 months, 15) zero vegetable or fruit consumption 6–23 months, 16) bottle feeding 0–23 months, and 17) infant feeding area graphs [1]. The WHO has defined each of the indicators to support consistency in IYCF practices terminology and measurement [1]. Indicators such as consumption of iron-rich or iron-fortified foods, age-appropriate breastfeeding, predominant breastfeeding under six months, and duration of breastfeeding were previously used but have been excluded from the latest recommendations [1].

IYCF practices are associated with parental, family, social, and policy level factors. Some common factors include parental age, education, employment and wealth status, and supportive policies (and their implementation) for working parents [4–6]. However, parental education is a particularly prominent factor because education increases health seeking behavior [7], decreases morbidity [8] and mortality [9], and fosters good health [10]. Education also leads to an uptake of better care practices [11] and can bring about positive behavioral change that can contribute to good health [12]. Furthermore, parental education is associated with the overall nutritional status and well-being of their children [13].

In Bangladesh, 34% of children 6–23 months of age are fed in accordance with the recommended IYCF practices [14]. In addition, 65% of children under the age of six months are exclusively breastfed [14]. Bangladesh has achieved commendable success in reducing child undernutrition [15]. This happened despite the absence of any strong nationwide nutrition programs and interventions [16]. Researchers showed that this achievement was primarily due to nutrition-sensitive factors and an improvement in overall socioeconomic status, where parental education is considered one of the major contributors [16–19]. Despite expected positive influence of parental education on IYCF practices, existing evidence from literature shows some incongruous association between parents’ education and their IYCF practices in Bangladesh. For example, Al Mamun et al. (2022) [20] found that exclusive breastfeeding was higher among mothers with high educational attainments compared to illiterate mothers whereas Hossain et al. (2018) [21] found that highly educated mothers had lower odds of exclusive breastfeeding than their counterparts. Furthermore, the practices of providing breast milk or milk products and ensuring at least four food groups and minimum meal frequency among mothers who completed at least secondary education level remained the same (47.5%) both in 2007 and 2017 [14, 22] despite improvements in maternal literacy rate. Moreover, the rate of bottle-feeding practice and providing infant formula was higher among educated mothers compared to non- and less educated mothers as shown in two nationally representative reports [23, 24]. Therefore, a systematic review could help elucidate the impact of parental education on IYCF practices based on available evidence in Bangladesh.

This study could be useful to understand the need of and formulate IYCF interventions specific for parents with different educational attainments. The findings of this review could help inform IYCF policymaking not only in Bangladesh but also in countries with similar context in Southeast Asia and other lower middle-income countries where an educational transition is ongoing.

### Theoretical basis

Theories help explain the mechanism of how parental education could influence IYCF practices. For instance, social cognitive theory asserts that human behavior, cognitive factors, and environmental factors influence each other through ongoing, reciprocal interactions [25]. In the context of IYCF, mothers/parents who have more knowledge on and positive attitudes toward IYCF recommendations would be expected to engage in recommended IYCF practices, given that they have the necessary skills, sufficient self-efficacy, and are supported by social norms. Education can play a significant role in strengthening cognitive and behavioral factors. For instance, institution-based formal education can greatly influence a person’s cognitive factors by shaping knowledge, attitudes, and expectations of behaviors such as IYCF [26]. Similarly, education helps individuals develop practical and intellectual skills and apply knowledge, cultivating their sense of self-efficacy [27, 28]. These cognitive and behavioral factors of an individual could subsequently influence other people and shape social norms [25]. By increasing one’s community access and influence on others, education could further influence environmental factors [29]. According to social cognitive theory, one would thus anticipate mothers/parents with higher levels of education to demonstrate better IYCF practices than less educated mothers/parents.

A mother’s cognitive, behavioral, and environmental factors are important to consider when examining IYCF practices. However, they may be insufficient, particularly in cases where higher education is correlated with poor IYCF. Positioning the relationship between (parental) education and IYCF practices within models of social determinants of health can provide valuable insights. The Dahlgren-Whitehead model [30] is composed of four interdependent layers that illustrate the main influences on health: individual lifestyle factors; social and community networks; living and working conditions; and general socioeconomic, cultural, and environmental conditions. Education is one aspect of a person’s material and social conditions in which they live and work and can greatly impact the individual’s lifestyle. While education can certainly have protective potential in achieving optimal IYCF (as suggested by the theoretical perspectives discussed above), higher levels of (parental) education may interrelate with other health determinants in such a way that hinders proper IYCF practices. For example, an educated mother may have a sound understanding of IYCF practices but is unable to fully apply her knowledge due to her work environment and long working hours. As such, higher levels of parental education may reduce barriers to IYCF but are unlikely to remove them completely. In circumstances in which a higher education status gives rise to new IYCF barriers, the most rational-seeming option for parents may be the one that goes against individual knowledge and professional advice to protect the overall wellbeing of the family (i.e., remaining employed). Thus, even though higher education may be a protective factor at the individual level, the interconnected role that educational status plays with socioeconomic and environmental conditions may render parental education a risk factor for IYCF.

While the propositions of social cognitive theory imply the positive impact of education on maternal child feeding behavior, examining education and IYCF through multilevel models of health determinants highlights that behavioral outcomes could be unpredictable due to barriers at various structural levels.

## Methods

This review is conducted following the Preferred Reporting Items for Systematic Review and Meta-Analysis (PRISMA) guideline [31] and is registered in PROESPERO (reg no: CRD42022355465).

### Data sources and search strategy

A search string was developed to find relevant articles in PubMed, Web of Science, Embase, and Google Scholar (provided as supplementary material). After conducting database searches, titles and abstracts of the records were exported to the software Covidence (https://www.covidence.org/) for removing duplicates and screening.

### Inclusion and exclusion criteria

We fitted the PECO criteria of systematic review as follows: population (P) = parents of infants and young children in Bangladesh, exposure (E) = higher levels of educational attainment of the parents, comparator (C) = parents with lower educational attainments compared to their counterparts, and outcome (O) = IYCF practices according to international recommendations [1].

Following the search strategy, any relevant records published from January 1980 to August 2022 were collected and screened for inclusion. This timeframe was chosen to allow the inclusion of as many relevant records as possible. Preliminary and manual searching found no relevant records before 1980. We considered following inclusion criteria: 1) participants: children’s mother and/or father; 2) exposure or intervention: education; 3) comparison: parents with different educational status; 4) outcome: all IYCF practices; 5) publication date: any articles published before August 2022; 6) language: English; 7) study design: quantitative study design such as cross-sectional, randomized controlled trial, cohort, and case control; 8) other documents: other documents, except original articles and thesis dissertations, were not considered; and 9) impact assessment: parental education was included in the statistical model and the comparison among different education groups was presented. We considered all the IYCF indicators as mentioned by the WHO [1].

### Selection process

Three reviewers (MAR, PB, and PS) independently conducted literature searching, screening of the titles and abstracts, and full text screening following the inclusion and exclusion criteria. At each stage, any controversy or disagreement regarding searching, inclusion, and exclusion of the records was independently settled by another reviewer (SS).

### Data extraction

At least two reviewers (MAR, PB, and PS) independently extracted the data from the selected articles. The data extraction checklist includes questions regarding: 1) author and publication, 2) study type, 3) study population and sample size, 4) geographical location, 5) type of association between education and IYCF practices (positive or negative), 6) comparison among different education groups as represented by odds ratio, regression coefficient, relative risks, or correlation coefficients, confidence intervals, and 7) level of significance (p-value). Data extraction was carried out using an Excel (Microsoft Corporation, 2022) spreadsheet. Finally, data was synthesized into a summary table.

### Quality assessment and addressing the risk of bias

Quality of the included studies was assessed with the Newcastle–Ottawa scale following the assessment criteria for cross-sectional, randomized control trail, and cohort studies (supplementary material) independently by at least two reviewers. Any discrepancy in decision making was independently resolved by another reviewer.

### Terminologies

We defined a “positive association” as the correlation of high parental education with good IYCF practices. A “negative association” was defined as high parental education being correlated with poor IYCF practices. Likewise, a “positively consistent association” was defined as a positive correlation between IYCF practices and level of parental education, and a “negatively consistent association” was defined as a negative correlation between these.

## Results

**Fig. 1:**
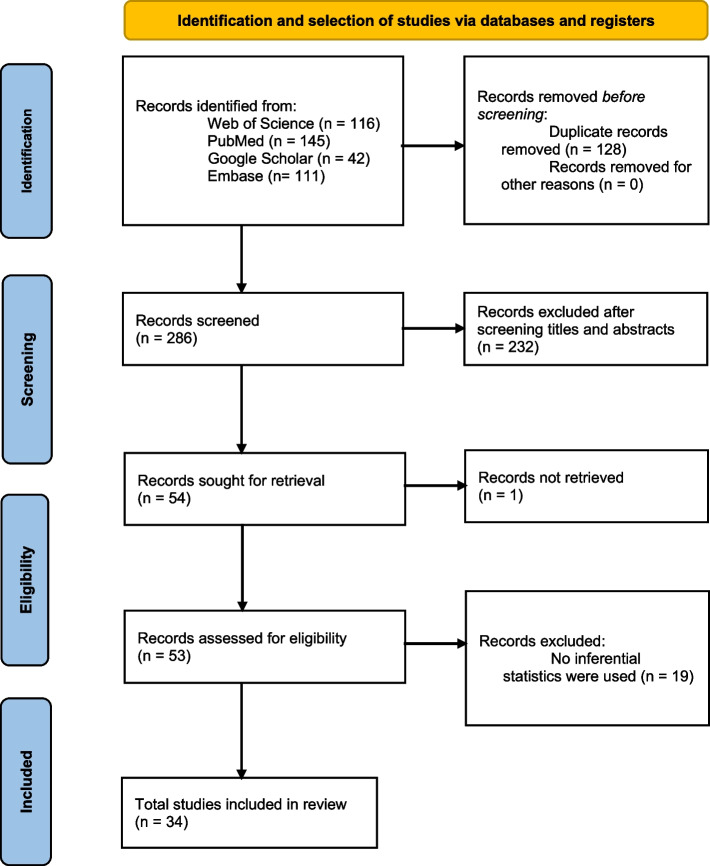
Selection process of the included studies

A total of 414 records were primarily retrieved through searching the databases. Out of these, 34 studies were included in this review. The PRISMA diagram showing the selection process of the included studies is presented in Fig. 1.

### Characteristics of the included studies

The included studies were published across a broad time range, with the oldest study having been published in 1981 [32]. Of the 34 included studies, 32 were cross-sectional studies, one was a retrospective longitudinal [33], and one was a randomized controlled trial [34]. Eleven studies considered parental (both parents) education [6, 21, 33, 35–42] and the remaining 23 studies considered only maternal education, all in relation to IYCF practices. Of the former eleven, nine studies separately considered father’s and mother’s education, and the remaining two studies [38, 39] considered both parents’ education together while analyzing their data. Twenty-four studies had a nationally representative sample size. Among the remaining ten, one was conducted in a defined area of north Bengal covering the districts Rangpur and Gaibandha [34] and another one in a defined area of Gaibandha [43]. Another study was conducted at a hospital in Dhaka [44]. Six studies were conducted at the sub-district level, and one at the district level (Rajshahi district). Among the included studies, the smallest sample size was 400 [20] and the largest sample size was 34,811 [45]. The characteristics of the included studies have been summarized in Table 1.

**Table 1:** Characteristics and quality of the included studies

Author, year	Study design	Data source	Caregiver type	Study population	Quality of the article^d^
Ahmed et al. (1999) [35]	Cross-sectional	Interview	Both parents	Four rural sub-districts	Good
Ahmmed & Manik (2021) [39]	Cross-sectional	BDHS^a^ 2004, 2007, 2011, and 2014	Both parents	National	Very good
Akter & Rahman (2010) [36]	Cross-sectional	BDHS^a^ 2004	Both parents	National	Very good
Akter & Rahman (2010) [46]	Cross-sectional	BDHS^a^ 2004	Mother	National	Very good
Akter et al. (2016) [40]	Cross-sectional	BDHS^a^ 2011	Both Parents	National	Good
Al Mamun et al. (2022) [20]	Cross-sectional	Interview	Mother	Suborno Char, Noakhali	Very good
Ali et al. (2019) [47]	Cross-sectional	Interview	Mother	20 sub-districts of north and north-east Bangladesh	Very good
Basnet et al. (2020) [48]	Cross-sectional	Alive & Thrive baseline data 2010	Mother	20 sub districts of Bangladesh	Very good
Blackstone & Sanghvi (2018) [45]	Cross-sectional	BDHS^a^ 2011, 2014	Mother	National	Very good
Campbell et al. (2016) [34]	Randomized controlled trial	Interview	Mother	Gaibandha and Rangpur	High quality
Chowdhury et al. (2016) [37]	Cross-sectional	BDHS^a^ 2011	Both parents	National	Very good
Dintyala (2020) [49]	Cross-sectional	BDHS^a^ 2014	Mother	National	Very good
Giashuddin & Kabir (2004) [50]	Cross-sectional	National survey^b^	Mother	National	Very good
Hasan et al. (2020) [44]	Cross-sectional	Interview	Mother	Dhaka, Bangladesh	Very good
Hossain et al. (2018) [21]	Cross-sectional	BDHS^a^ 2014	Mother	National	Very good
Islam et al. (2019) [6]	Cross-sectional	BDHS^a^ 2014	Both parents	National	Very good
Islam et al. (2019) [51]	Cross-sectional	BDHS^a^ 2014	Mother	National	Very good
Jain & Bongaart (1981) [32]	Cross-sectional	World Fertility Surveys 1976	Mother	National	Very good
Kabir et al. (2012) [5]	Cross-sectional	BDHS^a^ 2007	Mother	National	Very good
Karim et al. (2019) [52]	Cross-sectional	BDHS^a^ 2014	Mother	National	Very good
Khan et al. (2020) [41]	Cross-sectional	BDHS^a^ 2011, 2014	Both parents	National	Good
Khan et al. (2022) [53]	Cross-sectional	BDHS^a^ 2014	Mother	National	Very good
Mihrshahi et al. (2010) [54]	Cross-sectional	BDHS^a^ 2004	Both parents	National	Very good
Na et al. (2018) [42]	Cross-sectional	BDHS^a^ 2004–2014	Both parents	National	Very good
Nguyen et al. (2013) [55]	Cross-sectional	Interview	Mothers	20 subdistricts or upazilas	Very good
Rahman et al. (2011) [56]	Cross-sectional	BDHS^a^ 2007	Mother	National	Very good
Rahman et al. (2020) [33]	Retrospective longitudinal	HDSS of Icddr,b^c^	Mother (adolescent)	Matlab, Bangladesh	High risk study
Rana et al. (2020) [57]	Cross-sectional	Interview	Mother	Rajshahi district	Very good
Sakib et al. (2021) [58]	Cross-sectional	BDHS^a^ 2017–18	Mother	National	Good
Sen et al. (2020) [38]	Cross-sectional	BDHS^a^ 2004, 2007, 2011, 2014	Both parents	National	Very good
Senarath et al. (2012) [59]	Cross-sectional	BDHS^a^ 2007	Mother	National	Very good
Shahjahan et al. (2012) [60]	Cross-sectional	BDHS^a^ 2007	Mother	National	Very good
Sundaram et al. (2013) [43]	Cross-sectional	RCT data of Jivita1 trail	Mother	Gaibandha, Bangladesh	Very good
Tariqujjaman et al. (2022) [61]	Cross-sectional	BDHS^a^ 2017–18	Mother	National	Very good

^a^*BDHS* Bangladesh Demographic and Health Survey

^b^*National survey* Surveillance on Breastfeeding and Weaning Situation and Child and Maternal Health in Bangladesh

^c^Health & Demographic Surveillance System, International Center for Diarrheal Diseases Research, Bangladesh

^d^Quality was assessed by Newcastle–Ottawa Scale (NOS) (detailed scoring is provided as supplementary file)

After data extraction, the association between parental education and IYCF practices is summarized in Table 2.

**Table 2:** Association between parental education & child feeding in Bangladesh according to included articles

Author, year	IYCF component	Educational attainment	Association	Confidence interval	*P*-value	Type of analysis	Variables adjusted for	Association
Ahmed et al. (1999) [35]	Colostrum feeding	**Maternal**					Unadjusted	Colostrum feeding was positively associated with 1–5 years of parental education but negatively associated with ≥ 6 years of parental education compared to parents with no education. The associations were not statistically significant
		0 year of schooling^a^	1.00	Not mentioned	-	Logistic regression		
		1–5 years of schooling	1.27		> 0.10			
		≥ 6 years of schooling	0.91		> 0.10			
		**Paternal**						
		0 year of schooling^a^	1.00	Not mentioned	-	Logistic regression		
		1–5 years of schooling	1.02		> 0.10			
		≥ 6 years of schooling	0.70		> 0.10			
	Exclusive breastfeeding	**Maternal**					Unadjusted	Exclusive breastfeeding was negatively associated with parental education
		0 year of schooling^a^	1.00	Not mentioned	-	Logistic regression		
		1–5 years of schooling	0.72		< 0.10			
		≥ 6 years of schooling	0.49		< 0.01			
		**Paternal**						
		0 year of schooling^a^	1.00	Not mentioned	-	Logistic regression		
		1–5 years of schooling	0.92		> 0.10			
		≥ 6 years of schooling	0.91		> 0.10			
Ahmmed & Manik (2021) [39]	Early Initiation of breastfeeding	**Parental**					Year, Sex of child, mode of delivery, household member, exposure to media, birth order, type of birth, mother’s age at first birth, mother’s age at first marriage, delivery facility, BMI, place of residence, division, wealth index, mother’s working status	Children whose parents were educated were more likely to early initiation of breastfeeding than the children whose parents were uneducated
		Both uneducated^a^	1.00	-	-	Multilevel logistic regression		
		Any one educated	1.07	0.97, 1.18	0.182			
		Both educated	1.14	1.04, 1.26	0.007			
Akter & Rahman (2010) [36]	Breastfeeding cessation	**Maternal**					Age of mother, age at marriage, sex of child, parity, contraceptive use, delivery status, place of residence, division/region, education level of husband, work status of respondent, religion	Likelihood of breastfeeding cessation increased with an increase in maternal education
		No education	0.79	0.68, 0.92	< 0.01	Cox’s proportional hazard model		
		Primary	0.82	0.71, 0.94	< 0.01			
		Secondary	0.89	0.78, 1.02	< 0.05			
		Higher^a^	1.00	-	-			
		**Paternal**					Age of mother, maternal education, age at marriage, sex of child, parity, contraceptive use, delivery status, place of residence, division/region, work status of respondent, religion	Paternal education was not associated with breastfeeding cessation
		No education	1.00	0.92, 1.08	> 0.1	Cox’s proportional hazard model		
		Primary	1.00	0.93, 1.08	> 0.1			
		Secondary and higher^a^	1.00	-	-			
Akter & Rahman (2010) [46]	Breastfeeding cessation	**Maternal**					Residence, age at marriage, division/region, religion, work status, mother's age, parity, use of contraceptives	Likelihood of breastfeeding cessation increased with an increase in maternal education
		Illiterate	0.76	0.67, 0.87	0.00	Cox’s proportional hazard model		
		Primary	0.82	0.72, 0.94	0.00			
		Secondary	0.87	0.77, 0.98	0.03			
		Higher^a^	1.00	-	-			
Akter, et al. (2016) [40]	Immediate Initiation of breastfeeding	**Maternal**					Unadjusted	The odds of immediate breastfeeding were higher among educated parents
		Secondary^a^	1.00	-	Not mentioned	Logistic regression		
		Primary	0.88	0.63, 1.22				
		No education	0.79	0.57, 1.10				
		**Paternal**						
		Secondary^a^	1.00	-	Not mentioned	Logistic regression		
		Primary	0.97	0.74, 1.26				
		No education	0.88	0.68, 1.13				
Al Mamun et al. (2022) [20]	Exclusive breastfeeding	**Maternal**					Religion, mother’s age, mother’s level of education, type of delivery of the child, birth rank of the child, colostrum feeding of the child, breastfeeding initiation, frequency of breastfeeding, household food insecurity level	Likelihood of exclusive breastfeeding is higher among mothers with various levels of education compared with illiterate mothers
		Illiterate^a^	1.00	-	-	Logistic regression		
		Primary	3.36	1.71, 6.60	0.00			
		SSC/Dakhil	1.96	0.97, 3.95	0.06			
		HSC/Alim	1.82	0.53, 6.30	0.34			
		Graduate	4.41	1.00, 19.42	0.05			
		Madrasha education^*^	5.08	1.31, 19.72	0.02			
	Timely initiation of complementary feeding	**Maternal**					Mother’s age, mother’s level of education, birth rank of the child, colostrum feeding of the child, breastfeeding initiation, frequency of breastfeeding, household food insecurity level	Compared to illiterate mothers, mothers with SSC/Dakhil and HSC/Alim were less likely to have a timely initiation of complementary feeding and mothers with primary, graduate and madrasa education were more likely to have a timely initiation of complementary feeding
		Illiterate^a^	1.00	-	-	Logistic regression		
		Primary	1.18	0.49, 2.84	0.71			
		SSC/Dakhil	0.71	0.29, 1.76	0.46			
		HSC/Alim	0.65	0.10, 4.12	0.65			
		Graduate	1.35	0.12, 14.80	0.81			
		Madrasha education	2.91	0.38, 22.34	0.31			
Ali et al. (2019) [47]	Minimum dietary diversity (dietary diversity score)	**Maternal**					Sex of child, mothers’ age, mothers’ employment status, household monthly expenditure on food, household food security status, wealth quintile	Mothers with no education had more risk of not achieving minimum dietary diversity compared with counterparts
		No education^a^	1.00^b^	Not mentioned	-	Linear regression		
		Primary incomplete	0.02^b^		0.72			
		Primary to secondary	0.15^b^		< 0.05			
Basnet et al. (2020) [48]	Exclusive breastfeeding	**Maternal**					Maternal knowledge, nutritional status, mental well-being, decision-making capacity, employment, support in chores, perceived instrumental support	Maternal education had positive associations with exclusive breastfeeding
		1–5 years schooling^a^	1.00	Not mentioned	-	Multiple logistic regression		
		No schooling	1.36		≥ 0.05			
		6–9 years schooling	1.00		≥ 0.05			
		10–12 years schooling	1.16		≥ 0.05			
	Minimum meal frequency	**Maternal**					Maternal knowledge, nutritional status, mental well-being, decision-making, employment, support in chores, perceived instrumental support	Maternal education had positive associations with minimum meal frequency
		1–5 years schooling^a^	1.00	Not mentioned	-	Multiple logistic regression		
		No schooling	1.18		≥ 0.05			
		6–9 years schooling	0.95		≥ 0.05			
		10–12 years schooling	1.18		≥ 0.05			
	Dietary diversity	**Maternal**					Maternal knowledge, nutritional status, mental well-being, decision-making capacity, employment, support in chores, perceived instrumental support	Negative association were between dietary diversity and no schooling
		1–5 years schooling	Ref^b^	Not mentioned	-	Multiple linear regression		
		No schooling	-0.12^b^		≥ 0.05			
		6–9 years schooling	0.061^b^		≥ 0.05			
		10–12 years schooling	0.26^b^		≥ 0.05			
Blackstone & Sanghvi (2018) [45]	Minimum dietary diversity for 6-23 m children in 2011	**Maternal**					Wealth index, frequency of television watching, frequency of newspaper reading, decision making, place of delivery, number of antenatal visits, employment, region of residence	The minimum dietary diversity was positively associated with the increase in education levels of mothers in 2011
		No education^a^	1.00	-	-	Logistic regression		
		Primary education	1.36	0.94, 2.00	≥ 0.05			
		Secondary + education	1.91	1.25, 2.63	< 0.01			
	Minimum dietary diversity for 6-23 m children in 2014	**Maternal**					Wealth index, frequency of television watching, frequency of newspaper reading, decision making, place of delivery, number of antenatal visits, employment, region of residence	The minimum dietary diversity was positively associated with the increase in education levels of mothers in 2014
		No education^a^	1.00	-	-	Logistic regression		
		Primary education	1.36	0.93, 1.98	≥ 0.05			
		Secondary + education	2.28	1.58, 3.27	< 0.001			
	Complementary feeding for 18-23 m children in 2011	**Maternal**					Wealth index, frequency of television watching, frequency of newspaper reading, decision making, place of delivery, number of antenatal visits, employment, region of residence	The odds of complementary feeding among children were higher if maternal education level was higher in 2011
		No education^a^	1.00	-	-	Logistic regression		
		Primary education	1.45	0.80, 2.66	≥ 0.05			
		Secondary + education	1.53	0.93, 3.13	≥ 0.05			
	Complementary feeding for 18-23 m children in 2014	**Maternal**					Wealth index, frequency of television watching, frequency of newspaper reading, decision making, place of delivery, number of antenatal visits, employment, region of residence	The odds of complementary feeding among children were higher if maternal education level was higher in 2014
		No education^a^	1.00	-	-	Logistic regression		
		Primary education	1.25	0.71, 2.21	≥ 0.05			
		Secondary + education	2.58	1.49, 4.44	< 0.01			
Campbell et al. (2016) [34]	Minimum dietary diversity from home food at age 18 months	**Maternal**					Living standard index of socioeconomic status, household food insecurity score, sex	The probability of feeding minimum dietary diversity from home food at age 18 months was higher among mothers with higher educational levels
		None^a^	1.00	-	Not mentioned	Logistic regression		
		1–9 years	1.63	1.40, 1.90				
		SSC passed	2.51	1.83, 3.46				
		≥ 11 years	3.72	2.72, 5.10				
Chowdhury et al. (2016) [37]	Adequate complementary feeding practices	**Maternal**					Children age(months), sex of child, father’s education, father’s employment status, socioeconomic status, watching television, listening radio, reading newspaper/magazine, food insecurity, place of residence, region of residence	Adequate complementary feeding practices were higher among illiterate mothers compared to their literate counterparts
		Illitterate	1.48	0.46, 4.69	0.51	Logistic regression		
		Literate^a^	1.00	-	-			
		**Paternal**					Children age(months), sex of child, mother’s education, father’s employment status, socioeconomic status, watching television, listening radio, reading newspaper/magazine, food insecurity, place of residence, region of residence	Adequate complementary feeding practices were higher among illiterate fathers compared to their illiterate counterparts
		Illitterate	0.32	0.11, 0.95	0.04	Logistic regression		
		Literate^a^	1.00	-	-			
Dintyala (2020) [49]	Exclusive breastfeeding at 6 months	**Maternal**					Number of ANC visits, mother’s age, mother’s age, wealth quintile, place of delivery, postnatal breastfeeding counseling, ANC by medically trained persons	Compared to the mothers with higher education, mothers with primary & secondary education had greater odds of exclusive breastfeeding but fewer odds of mothers with no education
		No education	0.77	0.47, 1.24	0.28	Logistic regression		
		Primary	1.16	0.78, 1.71	0.47			
		Secondary	1.71	1.22, 2.38	0.00			
		Higher^a^	1.00	-	-			
Giashuddin & Kabir (2004) [50]	Stop breastfeeding	**Maternal**					Maternal age, place of residence, economic status, delivery assistance, preceding birth interval	Mothers with at least secondary level of education were more likely to stop breastfeeding than less or uneducated mothers
		No education^a^	1.00^c^	-	Not mentioned	Cox’s regression model		
		Primary	0.96^c^	0.84, 1.09				
		Secondary	1.19^c^	1.01, 1.28				
		Higher	1.25^c^	1.12, 1.86				
Hasan et al. (2020) [44]	Early Initiation of breastfeeding	**Maternal**					Maternal age, maternal occupation, family income, live birth, types of delivery, ANC check-up, pre-lacteal feeds given, counseling before delivery, baby’s birth weight, skin-to-skin contact after delivery	Mothers' education level significantly increased the likelihood of early initiation of breastfeeding among mothers
		Illiterate^a^	1.00	-	-	Multiple logistic regression		
		Primary	2.82	1.19, 6.67	0.019			
		SSC or above	4.04	1.59, 10.26	0.003			
Hossain et al. (2018) [21]	Exclusive breastfeeding	**Maternal**					Region, mother’s age, father’s education, mothers’ occupation, fathers’ occupation, mass media access, BMI of mother, total children ever born, delivery mode for last pregnancy, delivery place, antenatal care, postnatal care for mothers, breastfeeding counseling during first two days, current age of children	Compared to mothers with higher education level, relatively less educated mothers were more likely to exclusively breastfeed their children
		Illiterate	1.87	0.73, 4.76	0.19	Binary multivariable logistic regression		
		Primary	2.28	1.05, 4.93	0.04			
		Secondary	1.75	0.95, 3.24	0.07			
		Higher^a^	1.00	-	-			
		**Paternal**					Region, mother’s age, mothers’ education, mothers’ occupation, fathers’ occupation, mass media access, BMI of mother, total children ever born, delivery mode for last pregnancy, delivery place, antenatal care, postnatal care for mothers, breastfeeding counseling during first two days, current age of children	Compared to fathers with higher education, fathers with secondary or no education had higher odds of achieving exclusive breastfeeding in contrast to fathers with primary level of education
		Illiterate	1.16	0.54, 2.51	0.70	Binary multivariable logistic regression		
		Primary	0.98	0.50, 1.92	0.96			
		Secondary	1.24	0.69, 2.25	0.47			
		Higher^a^	1.00	-	-			
Islam et al. (2019) [6]	Breastfeeding continuation	**Maternal**					Division (administrative region), place of residence, father’s education level, religion, wealth index, mother’s body mass index, source of drinking water, toilet facility, household member, currently amenorrhea, currently abstaining, sex of child, child is twin	Breastfeeding duration was negatively associated with parental education
		Illiterate^a^	1.00	Not mentioned	-	Polytomous Logistic regression		
		Primary	1.03		0.80			
		Secondary	1.02		0.88			
		Higher	0.75		0.08			
		**Paternal**					Division (administrative region), place of residence, mother’s education level, religion, wealth index, mother’s body mass index, source of drinking water, toilet facility, household member, currently amenorrhea, currently abstaining, sex of child, child is twin	
		Illiterate^a^	1.00	Not mentioned	-	Polytomous Logistic regression		
		Primary	0.67		0.00			
		Secondary	0.68		0.00			
		Higher	0.73		0.03			
Islam et al. (2019) [51]	Early breastfeeding Initiation	**Maternal**					Division (administrative region), type of residence, place of delivery, Mother’s BMI, age at first marriage, wealth index, age at first birth, antenatal care visit, mode of delivery	The higher the level of mothers’ educational attainments, the lower the odds of early initiation of breastfeeding
		Uneducated^a^	1.00	-	-	Multivariable logistic regression		
		Primary	0.87	0.70, 1.09	0.22			
		Secondary	0.81	0.66, 0.99	0.05			
		Higher	0.58	0.44, 0.78	0.00			
Jain & Bongaarts (1981) [32]	Breastfeeding continuation	**Maternal**					Age, parity, infant deaths, residence, sex of child, workplace of wife	Highly educated mothers breastfed their children for a shorter duration
		Not highly educated^a^	1.00^b^	Not mentioned	Not mentioned	Multiple linear regression		
		Highly educated	-5.99^b^					
Kabir et al. (2012) [5]	Not introducing solid, semi solid, and soft foods to infants 6–8 months	**Maternal**					Gender of baby, age of child, birth order, preceding birth interval, diarrhea, ARI (acute respiratory infection), maternal age, maternal age at child’s birth, mother’s literacy, mother’s working status, mother’s BMI, mother’s religion, father’s education, father’s occupation, marital status, source of drinking water, household wealth index, reads newspaper or magazine, listens to radio, watches television, mode of delivery, type of delivery assistance, antenatal clinic visit, timing of postnatal checkup, residence, geographical region	Mothers with higher educational attainments were more likely to introduce solid, semi solid & soft foods to their children of 6–8 months of age
		Secondary & above^a^	1.00	-	-	Logistic regression		
		Primary	2.31	1.07, 4.96	0.03			
		No education	2.14	1.08, 4.23	0.03			
	Not meeting minimum dietary diversity among children aged 6–23 months	**Maternal**					Gender of baby, age of child, birth order, preceding birth interval, diarrhea, ARI (acute respiratory infection), maternal age, maternal age at child’s birth, mother’s literacy, mother’s working status, mother’s BMI, mother’s religion, father’s education, father’s occupation, marital status, source of drinking water, household wealth index, reads newspaper or magazine, listens to radio, watches television, mode of delivery, type of delivery assistance, antenatal clinic visit, timing of postnatal checkup, residence, geographical region	Mothers with higher educational attainments were more likely to attain minimum dietary diversity for their children aged 6–23 months of age
		Secondary & above^a^	1.00	-	-	Logistic regression		
		Primary	1.41	1.03, 1.94	0.03			
		No education	1.69	1.14, 2.54	0.01			
	Not meeting the minimum meal frequency among children aged 6–23 months	**Maternal**					Gender of baby, age of child, birth order, preceding birth interval, diarrhea, ARI (acute respiratory infection), maternal age, maternal age at child’s birth, mother’s literacy, mother’s working status, mother’s BMI, mother’s religion, father’s education, father’s occupation, marital status, source of drinking water, household wealth index, reads newspaper or magazine, listens to radio, watches television, mode of delivery, type of delivery assistance, antenatal clinic visit, timing of postnatal checkup, residence, geographical region	Mothers with higher educational attainments were more likely to meet minimum meal frequency for their children aged 6–23 months of age
		Secondary & above^a^	1.00	-	-	Logistic regression		
		Primary	1.26	0.88, 1.82	0.21			
		No education	1.70	1.09, 2.67	0.02			
	Not meeting minimum acceptable diet	**Maternal**					Gender of baby, age of child, birth order, preceding birth interval, diarrhea, ARI (acute respiratory infection), `maternal age, maternal age at child’s birth, mother’s literacy, mother’s working status, mother’s BMI, mother’s religion, father’s education, father’s occupation, marital status, source of drinking water, household wealth index, reads newspaper or magazine, listens to radio, watches television, mode of delivery, type of delivery assistance, antenatal clinic visit, timing of postnatal checkup, residence, geographical region	Mothers with higher educational attainments were more likely to meet a minimum acceptable diet for their children
		Secondary & above^a^	1.00	-	-	Logistic regression		
		Primary	1.36	1.01, 1.84	0.05			
		No education	1.73	1.20, 2.49	0.003			
Karim et al. (2019) [52]	Early Breastfeeding Initiation	**Maternal**					Mother’s age at childbirth, mother’s religion, wealth index, place of residence, division, birth order of child, number of ANC visits, mode of delivery, place of delivery, child’s size at birth, PNC within one hour of childbirth, skin-to-skin contact	Mothers with higher levels of education were less likely to initiate early breastfeeding compared to uneducated mothers
		No education^a^	1.00	-	-	Multivariable logistic regression		
		Primary	0.96	0.70, 1.32	0.79			
		Secondary or higher	0.97	0.71, 1.32	0.86			
Khan et al. (2020) [41]	Breastfeeding termination	**Maternal**					Survey year, heard about family planning, division, child’s sex, maternal age at childbirth, fathers’ education, maternal working status, contraceptive use, maternal malnutrition, birth order, wealth index, religion, exposure to media, and place of residence	Educated mothers were more likely to terminate breastfeeding their children earlier
		No education^a^	1.00	Not mentioned	-	Cox’s proportional hazard model		
		Primary education	1.41		< 0.05			
		Secondary or higher	1.70		< 0.001			
		**Paternal**					Survey year, heard about family planning, division, child’s sex, maternal age at childbirth, mothers’ education, maternal working status, contraceptive use, maternal malnutrition, birth order, wealth index, religion, exposure to media, and place of residence	The risk of breastfeeding termination was higher among children with paternal secondary or higher education and lower among with paternal primary education compared to their uneducated counterparts
		No education^a^	1.00	Not mentioned	≥ 0.05	Cox’s proportional hazard model		
		Primary education	0.94		≥ 0.05			
		Secondary or higher	1.06		≥ 0.05			
Khan et al. (2022) [53]	Minimum dietary diversity (MDD)	**Maternal**					Number of HH member, age of HH head, sex of HH head, maternal occupation, maternal malnutrition, sex of children, age of children, maternal age at childbirth, morbidity, maternal decision making, place of residence, wealth index, paternal occupation, birth order, religion, exposure to media, ANC visit, PNC Visit	Maternal education levels were positively associated with minimum dietary diversity of their children
		No education^a^	1.00	-	-	Multilevel logistic regression		
		Primary	1.35	0.91, 2.00	< 0.05			
		Secondary	2.48	1.68, 3.67	< 0.05			
		Higher	3.86	2.40, 6.20	< 0.05			
	Minimum meal frequency (MMF)	**Maternal**					Number of HH member, age of HH head, sex of HH head, maternal occupation, maternal malnutrition, sex of children, age of children, maternal age at childbirth, morbidity, maternal decision making, place of residence, wealth index, paternal occupation, birth order, religion, exposure to media, ANC visit, PNC visit	Maternal education levels were positively associated with minimum meal frequency of their children
		No education^a^	1.00	-	-	Multilevel logistic regression		
		Primary	0.99	0.72, 1.37	< 0.05			
		Secondary	1.43	1.04, 1.98	< 0.05			
		Higher	1.82	1.18, 2.79	< 0.05			
	Minimum acceptable diet (MAD)	**Maternal**					Number of HH member, age of HH head, sex of HH head, maternal occupation, maternal malnutrition, sex of children, age of children, maternal age at childbirth, morbidity, maternal decision making, place of residence, wealth index, paternal occupation, birth order, religion, exposure to media, ANC visit, PNC visit	Maternal education levels were positively associated with minimum acceptable diet of their children
		No education^a^	1.00	-	-	Multilevel logistic regression		
		Primary	0.98	0.71, 1.35	< 0.05			
		Secondary	1.38	1.00, 1.90	< 0.05			
		Higher	1.72	1.13, 2.61	< 0.05			
Mihrshahi et al. (2010) [54]	Not timely initiation of breastfeeding	**Maternal**					Unadjusted	The odds of not timely initiation of breastfeeding decreased with an increase in parental education
		None^a^	1.00	-	-	Logistic regression		
		Primary	0.77	0.53, 1.12	≥ 0.05			
		Secondary or above	0.61	0.44, 0.84	< 0.01			
		**Paternal**						
		None^a^	1.00	-	-	Logistic regression		
		Primary	0.81	0.61, 1.07	≥ 0.05			
		Secondary or above	0.59	0.38, 0.92	< 0.05			
	Not exclusively breastfeeding	**Maternal**					Maternal working status, mother’s marital status, husband’s education, mother’s age, birth order of child, preceding birth interval, sex of child, age of child, place of delivery, type of delivery assistance, number of antenatal care visits, timing of postnatal checkups, mother’s BMI, mother reads newspaper, mother listens to radio, mother watches television, household wealth index number of categories of decision in which women have final say, region of residence, geographic region	Odds of not exclusively breastfeeding was higher among educated mothers compared to non-educated groups
		None^a^	1.00	-	-	Multiple logistic regression		
		Primary	2.23	1.32, 3.76	< 0.01			
		Secondary or above	1.82	0.94, 3.55	≥ 0.05			
	Bottle feeding practice	**Paternal**					Maternal working status, mother’s marital status, husband’s education, mother’s age, birth order of child, preceding birth interval, sex of child, age of child, place of delivery, type of delivery assistance, number of antenatal care visits, timing of postnatal checkups, mother’s BMI, mother reads newspaper, mother listens to radio, mother watches television, household wealth index number of categories of decision in which women have final say, region of residence, geographic region	Odds of bottle-feeding practices were increased with the increase in paternal education
		None^a^	1.00	-	-	Multiple logistic regression		
		Primary	1.06	0.73, 1.54	≥ 0.05			
		Secondary or above	2.17	1.30, 3.64	< 0.01			
	Not given timely complementary foods	**Maternal**					Unadjusted	The odds of not timely giving complementary foods decreased with an increase in maternal education
		None^a^	1.00	-	-	Multiple logistic regression		
		Primary	0.85	0.48, 1.50	≥ 0.05			
		Secondary or above	0.62	0.37, 1.06	≥ 0.05			
		**Paternal**						
		None^a^	1.00	-	-	Multiple logistic regression		
		Primary	0.57	0.35, 0.93	< 0.05			
		Secondary or above	0.59	0.29, 1.20	≥ 0.05			
Na et al. (2018) [42]	Introduction of solid, semi-solid, and soft foods	**Maternal**					Year, children’s age, birth order, birth interval, age-appropriate vaccination, maternal age, BMI, type of delivery assistance, antenatal care visits, father’s age, paternal education, household wealth, geographical region, rank of access to healthcare	Introduction of solid, semi-solid & soft foods was positively associated with maternal education
		No education	0.68	0.46, 1.03	0.07	Multivariable multilevel logistic regression		
		Primary	0.89	0.64, 1.24	0.49			
		Secondary or higher^a^	1.00	-	-			
		**Paternal**					Year, children’s age, birth order, birth interval, age-appropriate vaccination, maternal age, education, BMI, type of delivery assistance, antenatal care visits, father’s age, household wealth, geographical region, rank of access to healthcare	Compared to secondary or higher educated fathers, primary educated fathers had higher and uneducated fathers had lower odds of introducing solid, semi-solid, and soft foods
		No education	0.86	0.59, 1.24	0.41	Multivariable multilevel logistic regression		
		Primary	1.29	0.92, 1.80	0.14			
		Secondary or higher^a^	1.00	-	-			
	Minimum meal frequency	**Maternal**					Year, children’s age, birth order, birth interval, age-appropriate vaccination, maternal age, BMI, type of delivery assistance, antenatal care visits, father’s age, paternal education, household wealth, geographical region, rank of access to healthcare	Mothers with higher educational attainments were more likely to attain minimum meal frequency for their children
		No education	0.71	0.59, 0.86	< 0.001	Multivariable multilevel logistic regression		
		Primary	0.74	0.64, 0.85	< 0.001			
		Secondary or higher^a^	1.00	-	-			
		**Paternal**					Year, children’s age, birth order, birth interval, age-appropriate vaccination, maternal age, education, BMI, type of delivery assistance, antenatal care visits, father’s age, household wealth, geographical region, rank of access to healthcare	Fathers with higher education attainment had less likelihood of attaining minimum meal frequency for their children
		No education	0.92	0.78, 1.09	0.32	Multivariable multilevel logistic regression		
		Primary	1.03	0.89, 1.19	0.70			
		Secondary or higher^a^	1.00	-	-			
	Minimum dietary diversity	**Maternal**					Year, children’s age, birth order, birth interval, age-appropriate vaccination, maternal age, BMI, type of delivery assistance, antenatal care visits, father’s age, paternal education, household wealth, geographical region, rank of access to healthcare	Mothers with higher educational attainments were more likely to achieve minimum dietary diversity for their children
		No education	0.54	0.40, 0.73	< 0.001	Multivariable multilevel logistic regression		
		Primary	0.71	0.58, 0.86	< 0.01			
		Secondary or higher^a^	1.00	-	-			
		**Paternal**					Year, children’s age, birth order, birth interval, age-appropriate vaccination, maternal age, education, BMI, type of delivery assistance, antenatal care visits, father’s age, household wealth, geographical region, rank of access to healthcare	Fathers with higher educational attainments were more likely to achieve minimum dietary diversity for their children
		No education	0.76	0.59, 0.96	< 0.05	Multivariable multilevel logistic regression		
		Primary	0.87	0.73, 1.05	0.16			
		Secondary or higher^a^	1.00	-	-			
	Minimum acceptable diet	**Maternal**					Year, children’s age, birth order, birth interval, age-appropriate vaccination, maternal age, BMI, type of delivery assistance, antenatal care visits, father’s age, paternal education, household wealth, geographical region, rank of access to healthcare	Mothers with higher educational attainments were more likely to achieve minimum acceptable diet for their children
		No education	0.57	0.41, 0.78	< 0.01	Multivariable multilevel logistic regression		
		Primary	0.79	0.65, 0.97	< 0.05			
		Secondary or higher^a^	1.00	-	-			
		**Paternal**					Year, children’s age, birth order, birth interval, age-appropriate vaccination, maternal age, education, BMI, type of delivery assistance, antenatal care visits, father’s age, household wealth, geographical region, rank of access to healthcare	Fathers with higher educational attainments were more likely to achieve minimum acceptable diet for their children
		No education	0.74	0.57, 0.95	< 0.05	Multivariable multilevel logistic regression		
		Primary	0.87	0.71, 1.05	0.15			
		Secondary or higher^a^	1.00	-	-			
Nguyen et al. (2013) [55]	Dietary diversity (complementary feeding)	**Maternal**					Maternal dietary diversity, age, knowledge, self-perceived physical health, income, decision making in buying foods, food security, socioeconomic status, mother as household head, number of children, child’s sex	Maternal education was positively associated with achieving dietary diversity of children
		No schooling^a^	1.00^b^	-	-	Multivariable linear regression		
		Primary	0.11^b^	-0.08, 0.30	< 0.05			
		Secondary	0.20^b^	-0.13, 0.52	< 0.05			
		College or higher	0.80^b^	0.39, 1.22	< 0.001			
Rahman et al. (2011) [56]	Early breastfeeding initiation	**Maternal**					Mother’s age at birth, residence, sex of child, birth interval, wealth autonomy, antenatal care, frequency of mass media exposure, wealth index, delivery assistance	Maternal education was positively associated with early initiation of breastfeeding
		No education^a^	1.00	-	-	Logistic regression		
		Primary	1.40	0.28, 1.86	< 0.05			
		Secondary	1.80	0.94, 2.98	< 0.05			
Rahman et al. (2020) [33]	Exclusive breastfeeding	**Maternal**					Mother’s age at first birth, sex of child, religion, asset score, fathers’ education, repeated pregnancy, religion, area, place of delivery, mode of delivery, history of 4 + ANC	Compared to mothers with secondary or higher education, mothers with no education and mothers with primary education were more likely to exclusively breastfeed their children
		No education	1.02	0.63, 1.66	Not mentioned	Cox’s proportional hazard model		
		Primary	1.03	0.85, 1.24				
		Secondary or higher^a^	1.00	-				
		**Paternal**					Mother’s age at first birth, sex of child, religion, asset score, mothers’ education, repeated pregnancy, religion, area, place of delivery, mode of delivery, history of 4 + ANC	Exclusive breastfeeding increased for children whose father had primary level of education but decreased in case of uneducated fathers
		No education	0.89	0.76, 1.05	Not mentioned	Cox’s proportional hazard model		
		Primary	1.11	0.91, 1.34				
		Secondary or higher^a^	1.00	-				
Rana et al. (2020) [57]	Exclusive breastfeeding	**Maternal**					Maternal age, religion, delivery place, occupation, type of family, monthly income	Mothers with higher levels of education were more likely to achieve exclusive breastfeed compared to the uneducated mothers
		Illiterate^a^	1.00	-	-	Binary logistic regression		
		Primary	1.19	0.41, 3.55	0.75			
		Secondary & higher	1.44	0.60, 3.45	0.42			
Sakib et al. (2021) [58]	Early breastfeeding initiation	**Maternal**					Unadjusted	Compared to illiterate mothers, mothers with higher levels of education were less likely to initiate early breastfeeding for their children
		Illiterate^a^	-	-	-	Multinomial logistic regression		
		Primary	1.55	1.14, 2.11	< 0.01			
		Secondary	1.53	1.14, 2.06	< 0.01			
		Higher	1.50	1.08, 2.10	< 0.01			
Sen et al. (2020) [38]	Early breastfeeding initiation	**Parental**					Survey time, maternal age at birth, gender of newborn, area of residence, birth order, wanted index child, place of delivery, mode of delivery, ANC visits, wealth index, exposure of media, division	Early initiation of breastfeeding was higher for parents with higher educational attainments
		Both uneducated^a^	1.00	-	-	Binary logistic regression		
		Any one educated	1.11	1.03, 1.19	< 0.01			
		Both educated	1.12	1.03, 1.22	< 0.001			
Senarath et al. (2012) [59]	Not introduction of solid, semi-solid or soft food (6–8 months)	**Maternal**					Age of children, birth order, diarrhoea, mother’s age, mother working status, mother’s BMI, father’s occupation, reads newspaper, household wealth index, religion, antenatal clinic visits, geographical region,	Mothers with higher educational attainments were more likely to timely introduce solid, semi-solid & soft foods to their children compared to uneducated mothers
		Secondary and above^a^	1.00	-	-	Multiple logistic regression		
		Primary	2.31	1.07, 4.96	0.03			
		No education	2.14	1.08, 4.23	0.03			
	Inappropriate dietary diversity (6–23 months)	**Maternal**					Child’s age, acute respiratory infection, mother working status, mother’s height, mother’s BMI, decision making at household(maternal), household wealth index, reads newspaper, watches television, listen to radio, antenatal clinic visits, number of postnatal visits by public health midwives, timing of postnatal checkup, type of residence, geographical region,	Mothers with higher educational attainments were more likely to practice appropriate dietary diversity for their children than uneducated mothers
		Secondary and above^a^	1.00	-	-	Multiple logistic regression		
		Primary	1.41	1.03, 1.94	0.03			
		No education	1.70	1.14, 2.54	0.01			
	Inadequate meal frequency (6–23 months)	**Maternal**					Child’s age, mother working status, mother’s BMI, decision making at household(maternal), household wealth index, reads newspaper, listen to radio, watches television, exposure to media, antenatal clinic visits, antenatal home visits by public health midwives, type of residence, geographical region	Mothers with higher educational attainments were more likely to achieve adequate meal frequency for their children compared to uneducated mothers
		Secondary and above^a^	1.00	-	-	Multiple logistic regression		
		Primary	1.26	0.88, 1.82	0.21			
		No education	1.70	1.09, 2.67	0.02			
	Not meeting minimum acceptable diet (6–23 months)	**Maternal**					Child’s age, acute respiratory infection, mother working status, mother’s height, mother’s BMI, decision making at household(maternal), household wealth index, reads newspaper, listen to radio, exposure to media, antenatal clinic visits, antenatal home visits by public health midwives, type of residence, geographical region	Mothers with higher educational attainments were more likely to meet a minimum acceptable diet for their children compared to uneducated mothers
		Secondary and above^a^	1.00	-	-	Multiple logistic regression		
		Primary	1.36	1.01, 1.84	0.05			
		No education	1.73	1.20, 2.49	0.00			
Shahjahan et al. (2012) [60]	Early breastfeeding initiation	**Maternal**					Mother’s age, birth order, wealth index, religion, sex of child, antenatal checkup	Early initiation of breastfeeding was higher among mothers with higher educational attainments
		No education^a^	1.00	-	Not mentioned	Logistic regression		
		Primary	1.20	1.00, 1.40				
		Secondary	1.60	1.20, 2.20				
Sundaram et al. (2013) [43]	Pre-lacteal feeding or early neonatal feeding (ENF)	**Maternal**				Multiple logistic regression	Mother’s age, primigravidity, wealth quintile, perception of child size at birth, male child gender, behavior (normal suckling at birth), birth location, assistant present at birth, number of antenatal care visits, participation in BRAC microcredit program	Mothers who passed class nine were less likely to practice pre-lacteal feeding or early neonatal feeding compared to mothers who didn’t pass class.
		Passed class nine	0.8	0.70, 0.90	< 0.05			
		Did not pass class nine^a^	1.0	-	-			
		**Maternal**						
		Literate	1.0	0.90, 1.20	< 0.05			
		Illiterate^a^	1.0	-	-			
Tariqujjaman et al. (2022) [61]	Early initiation of breastfeeding	**Maternal**					Mothers’ age, age of children, sex of the children, type of place of residence, wealth index	Mothers with at least secondary education were less likely to practice early initiation of breastfeeding for their children compared to mothers with less than secondary education
		< secondary^a^	1.00	-	-	Generalized estimating equation model linked with log-binomial model		
		≥ secondary	0.84	0.72, 0.99	0.04			
	Exclusive breastfeeding	**Maternal**					Mothers’ age, age of children, sex of the children, type of place of residence, wealth index	Mothers who had at least secondary education were less likely to exclusively breastfeed their children compared to mothers with less than secondary level of education
		< secondary^a^	1.00	-	-	Generalized estimating equation model linked with log-binomial model		
		≥ secondary	0.89	0.65, 1.23	0.37			
	Continued breastfeeding at year 1	**Maternal**					Mothers’ age, age of children, sex of the children, type of place of residence, wealth index	Mothers who had at least secondary education were more likely to continue breastfeeding their children throughout year 1 compared to mothers with less than secondary education
		< secondary^a^	1.00	-	-	Generalized estimating equation model linked with log-binomial model		
		≥ secondary	1.04	0.99, 1.09	0.29			
	Introduction of solid semi-solid and soft foods	**Maternal**					Mothers’ age, age of children, sex of the children, type of place of residence, wealth index	Mothers who had at least secondary education were more likely to timely introduce solid, semi-solid & soft foods than their counterparts
		< secondary^a^	1.00	-	-	Generalized estimating equation model linked with log-binomial model		
		≥ secondary	1.04	0.90,1.21	0.90			
	Minimum dietary diversity	**Maternal**					Mothers’ age, age of children, sex of the children, type of place of residence, wealth index	Mothers who had at least secondary education were more likely to achieve minimum dietary diversity for their children than mothers with less than secondary education
		< secondary^a^	1.00	-	-	Generalized estimating equation model linked with log-binomial model		
		≥ secondary	1.85	1.51,2.27	< 0.001			
	Minimum meal frequency	**Maternal**					Mothers’ age, age of children, sex of the children, type of place of residence, wealth index	Mothers who had at least secondary education were more likely to achieve minimum meal frequency for their children than mothers with less than secondary education
		< secondary^a^	1.00	-	-	Generalized estimating equation model linked with log-binomial model		
		≥ secondary	1.02	0.97, 1.07	0.002			
	Minimum acceptable diet	**Maternal**					Mothers’ age, age of children, sex of the children, type of place of residence, wealth index	Mothers who had at least secondary education were more likely to achieve minimum acceptable diet for their children than mothers with less than secondary education
		< secondary^a^	1.00	-	-	Generalized estimating equation model linked with log-binomial model		
		≥ secondary	1.81	1.47–2.22	< 0.001			
	Consumption of iron-rich or iron-fortified foods	**Maternal**					Mothers’ age, age of children, sex of the children, type of place of residence, wealth index	Mothers with equal or more than secondary education had higher odds of feeding iron rich/fortified foods compared to their counterparts
		< secondary^a^	1.00	-	-	Generalized estimating equation model linked with log-binomial model		
		≥ secondary	1.69	1.36, 2.10	< 0.001			

Depending on type of analysis, adjusted odds ratio (AOR) and adjusted hazard ratio (AHR) were considered to compare different education groups, otherwise indicated

^a^Reference category for comparison

^b^Regression coefficient

^c^Relative risk

Confidence intervals are at 95% level, otherwise indicated

^*^Madrasha education is a type of education with a focus on Islamic religion in Bangladesh

### Early initiation of breastfeeding

Twelve studies focused on the early initiation of breastfeeding [35, 38–40, 44, 51, 52, 54, 56, 58, 60, 61], and of them one focused on colostrum feeding [35]. Of these, eight studies found positive associations of education with the early initiation of breastfeeding [38–40, 44, 54, 56, 58, 60]. These associations were positively consistent with the level of educational attainment, i.e., the association (e.g., odds ratio) increased with increasing level of education, except in the study of Sakib et al. (2021) [58]. Four studies [38–40, 54] considered both parents’ education and found that early initiation of breastfeeding is higher among comparatively highly-educated parents. Among these four studies, two did not mention the level of parental education, and the margin when differentiating between the educated and uneducated group in terms of their educational attainment was uncertain [38, 39]. For example, these studies presented the likelihood of early initiation of breastfeeding when any one or both parents were educated versus when both parents were uneducated. The remaining two studies categorized the education level as ‘no education’, ‘primary education’, and ‘ ≥ secondary education’ [40, 54].

Three studies found negative associations between maternal education and early initiation of breastfeeding [51, 52, 61]. All three studies considered maternal education only. In all cases, mothers with lower education categories compared to their counterparts were considered as the reference group. Among these, one study found a negatively consistent association [51], another study found an inconsistent association [52] and the final study considered only two groups (< secondary and ≥ secondary education) and found a negative association [61]. Ahmed et al. (1999) [35] investigated the impact of parental education on colostrum feeding and found a positive association with 1–5 years of parental education. However, the association was negative when parental education was more than or equal to six years, compared to the parents with no education as the reference category.

### Exclusive breastfeeding

Nine studies investigated the impact of parental education on exclusive breastfeeding status of infants [20, 21, 33, 35, 48, 49, 54, 57, 61]. Among these, Ahmed et al. (1999) [35], Hossain et al., 2018 [21], and Rahman et al. (2020) [33] considered both the father’s and mother’s education. The remaining studies considered only maternal education. Four studies found positive associations between maternal education and exclusive breastfeeding [20, 54, 57, 61]. Compared to the reference category (illiterate mothers), the association was found to be positively consistent by Rana et al. (2020) [57], although Al Mamun et al. (2022) [20] found an inconsistent association among the comparison groups such as illiterate mothers, mothers with primary, secondary, higher secondary, graduation level, and Madrasha education.

Three studies found negative associations between parental education and exclusive breastfeeding [21, 35, 48]. Ahmed et al. (1999) [35] considered mothers with no education as the reference category whereas Hossain et al. (2018) [21] considered mothers with higher education as the reference category. Ahmed et al. (1999) [35] found that the association was negatively inconsistent for both mothers’ and fathers’ education. For example, compared to the parents with no education, parents with 1–5 and ≥ 6 years of education were less likely to breastfeed exclusively. Basnet et al. (2020) [48] found a negatively consistent association between years of maternal schooling and exclusive breastfeeding.

Rahman et al. (2020) [33] found that compared to mothers with secondary or higher education, exclusive breastfeeding increased for mothers with primary education (OR: 1.03, 95% CI: 0.89,1.23) and mothers with no education (OR: 1.02: 95% CI: 0.63,1.66). But in the case of fathers, the association is inconsistent. Compared to the fathers with secondary or higher education, exclusive breastfeeding increased for children whose fathers had primary level of education but decreased for fathers with no education.

The association did not follow any specific direction for the study conducted by Dintyala (2020) [49] who found that the odds of exclusive breastfeeding was higher for the mothers with primary and secondary education but lower for the mothers with no education when considering the mothers with higher education as reference category.

### Duration of breastfeeding

Seven studies investigated the impact of parental education on the breastfeeding duration [6, 32, 36, 41, 46, 50, 61]. Except the study of Tariqujjaman et al. (2022) [61], all the studies found a negative association. However, Tariqujjaman et al. (2022) [61] did not find the association statistically significant (OR = 1.06, 95% CI = 0.99, 1.03). Akter & Rahman (2010) [36] and Akter & Rahman (2010) [46] found that mothers’ education is negatively consistent in relation to the duration of breastfeeding. Compared to the mothers with higher education, mothers with no education, primary education, or secondary education had lower risk of breastfeeding cessation with statistically significant associations in all cases. Jain & Bongaarts (1981) [32] also found a similar association of mothers’ education with the breastfeeding duration. Compared to the mothers with no education, mothers with primary, secondary, or more education had shorter mean duration of breastfeeding.

Giashuddin & Kabir (2014) [50] found mothers with primary education had a slightly lower relative risk (RR = 0.96) of stopping breastfeeding than the mothers with no education. On the other hand, the risk of breastfeeding cessation was higher among the mothers with secondary (RR = 1.19) and higher (RR = 1.25) education. Islam et al. (2019) (6) also found that mothers with primary and secondary education had 3% and 2% higher likelihood of breastfeeding continuation, respectively, compared to the mothers with no education; however, the likelihood was found to be 25% lower for the mothers with higher education.

Parental (both father and mother) education was considered by Akter & Rahman (2010) [36] and Islam et al. (2019) [6]. In both cases, maternal education was negatively associated with breastfeeding duration; however, Akter & Rahman (2010) [36] found that fathers’ education had no impact (OR = 1.00 for all education categories with *p* > 0.01) on duration of breastfeeding, whereas Islam et al. (2019) [6] found a negative association between paternal education and breastfeeding continuation.

Khan et al. (2020) [41] investigated the tendency of breastfeeding termination among mothers with different levels of education and children’s fathers with different levels of education and found that the likelihood of early termination of breastfeeding was consistently positive with the increase in maternal education.

In case of the fathers, Khan et al. (2020) [41] found that, compared to the children of fathers with no education, children whose fathers had completed primary education are less likely to experience terminated breastfeeding earlier while children whose fathers had secondary or higher educational level are more likely to experience terminated breastfeeding earlier.

### Complementary feeding

Thirteen studies investigated the impact of parental education on complementary feeding practices [5, 20, 34, 37, 42, 45, 47, 48, 53–55, 59, 61]. Of these, three studies considered both father’s and mother’s education [37, 42, 54]. In general, all the studies found that parental education was positively associated with complementary feeding practices, such as ensuring introduction of semi-solid, solid, and soft foods at the age of 6–8 months, minimum dietary diversity, minimum meal frequency, and minimum acceptable diet. Tariqujjaman et al. (2022) [61] found that maternal education had no association with introduction of solid, semi-solid, and soft foods (OR = 1.0, 95% CI = 0.93,1.05) whereas Basnet et al. (2020) [48] found a negative association between maternal education and dietary diversity.

Meanwhile, Al Mamun et al. (2022) [20] found both positive and negative associations among groups with different educational attainment as compared with the reference group (illiterate mothers). For example, the odds of timely initiation of complementary feeding was higher among mothers with primary (OR = 1.18, 95% CI = 0.49, 2.84), graduate (OR = 1.35, 95% CI = 0.12, 14.80), and Madrasha education (OR = 2.91, 95% CI = 0.38, 22.34), but lower among the mothers with SSC/Dakhil (secondary level) (OR = 0.71, 95% CI = 0.29, 1.76) and HSC/Alim (higher secondary level) (OR = 0.65, 95% CI = 0.10, 4.12) education. A similarly positive association was found by Mihrshahi et al. (2010) [54]. Chowdhury et al. (2016) [37] found adequate complementary feeding to be positively associated with paternal education but negatively associated with maternal education. Nguyen et al. (2013) [55] and Basnet et al. (2020) [48] calculated dietary diversity as part of complementary feeding practices and found positive association in relation to maternal education.

### Bottle feeding, pre-lacteal feeding, and iron rich/fortified foods

One study investigated the association between parental education and bottle-feeding practices [54] and found that bottle feeding practices are more likely for children whose fathers had higher levels of education compared to fathers with no educational attainments. Tariqujjaman et al. (2022) [61] investigated if the provision of iron rich/fortified foods is associated with maternal education and found the association to be positive (OR = 1.09, 95% CI = 1.06, 1.12). Sundaram et al. (2013) [43] found that pre-lacteal feeding or early neonatal feeding was more prevalent among mothers who did not pass class nine compared to those who passed class nine. On the other hand, pre-lacteal feeding is almost the same among both illiterate and literate mothers.

### Quality of the included studies

For each of the included studies, detailed scoring for each item/criterion according to Newcastle–Ottawa Scale (NOS) is provided as supplementary material. For the 32 cross-sectional studies, the score ranged from 7 to 10 out of a total score of 10 (Table 1). Of them, four studies had a score between 7–8 which is considered good, and other 28 studies had a score between 9–10 which is considered very good according to NOS assessment criteria. For one randomized controlled trial and one cohort study the score was 8 and 6, respectively.

Apart from NOS assessment criteria, we also checked studies that reported how the multicollinearity issues were estimated/handled. Only five studies reported how the multicollinearity was estimated. Among them, two studies reported variance inflation factor (VIF) [47, 61] and three studies reported that standard error (SE) was used to estimate multicollinearity [5, 51, 56]. Regarding predictability of the statistical model, only two studies reported the R^2^ value. For Hossain et al. (2018) [21], R^2^ value of the statistical model was 0.885 whereas for Jain & Bongaarts (1981) [32], the value was 0.57. In four studies, the estimated association was not adjusted for other variables such as gender of the children, employment status, type of delivery, and household wealth index [35, 40, 54, 58]. In the study of Mihrshahi et al. (2010) [54] four IYCF indicators––including not timely initiation of breastfeeding, not exclusively breastfeeding, bottle feeding, and not timely complementary feeding––were considered; however, the estimated association was adjusted only for two outcome variables, including not exclusively breastfeeding and bottle-feeding practices.

## Discussion

This study explores the pattern of associations between IYCF practices and parental education in Bangladesh. We found that parental education was both positively and negatively associated with IYCF, depending on the different IYCF components. For example, comparing the reference category with others, parental education, in general, was found to be positively associated with complementary feeding status [5, 20, 34, 37, 42, 45, 47, 48, 53, 55, 59, 61], but negatively associated with breastfeeding related indicators [6, 21, 32, 35, 36, 41, 46, 50–52, 61]. However, some studies also found positive association between parental education and breastfeeding related indicators [20, 33, 38, 39, 44, 49, 57, 60].

In this review, 34 studies were included of which 24 studies analyzed the datasets from nationally representative surveys (e.g., Bangladesh Demographic and Health Survey). The remaining ten studies were conducted in different districts and sub-districts in Bangladesh, considering representative samples of their target population. Included studies were conducted in a wide range of periods extending from 1981 to 2022. Therefore, the evidence is substantial to draw a logical conclusion regarding the association between parental education and IYCF practices in Bangladesh, considering the representativeness of data.

A considerable socio-economic transition, especially an increase in literacy rate, has occurred in the last four decades in Bangladesh. The adult literacy rate was 29% in 1981, 35% in 1991, 47% in 2001, 59% in 2011, and 75% in 2020 [62]. Additionally, access to education, particularly for women, and female literacy rate have improved significantly [63]. Besides, notable empowerment of women has occurred through their growing employment rate within this period [64]. Apart from these, access to information through the printed, electronic, and social media has increased [45]. Despite these socio-economic changes, IYCF practices do not show a proportionate improvement over this time period, regardless of development not only in maternal education but also in household wealth quintile of mothers [14, 22]. In our review, for example, Jain & Bongaarts (1981) [32] found that the duration of breastfeeding was negatively associated with an increase in maternal education. Similar association was found by a recent study conducted by Islam et al. (2019) [6] where mothers with higher level of education were found to have less breastfeeding duration than mothers with no education.

A fluctuation is observed in IYCF practices in the last three decades [14]. The exclusive breastfeeding rate has increased to 65% in 2017–18 from 45.9% in 1993–94. However, our findings show that the odds of exclusive breastfeeding were higher for a mother with lower educational attainment than her counterparts [21, 35]. This implies that the impact of increasing literacy rate among women on breastfeeding is ambiguous. In addition, compared to no maternal education, higher educational attainment was found to be positively associated (OR = 2.17, 95% CI = 1.30, 3.64) with bottle feeding practices [54]. In contrast to exclusive breastfeeding and bottle feeding practices, parental education was found to be positively associated with complementary feeding of children over time according to available records [5, 20, 47].

Why parental education is positively associated with complementary feeding practices but negatively associated with breastfeeding-related indicators requires further investigation. Educated mothers are more likely to be employed and have control over resources and therefore have a say in family decision making. However, employment could sometimes be a barrier to optimum IYCF practices. For example, an employed mother is less likely to practice breastfeeding if she has to spend a longer period of time at the workplace [21, 49]. Hence, she could use bottle feeding as a proxy for breastfeeding, thereby shortening breastfeeding duration. Bottle feeding allows mothers to work and can be performed by someone other than the mother [65].

Education is a proxy indicator of socioeconomic position which could be related to exposure to advertisement and financial capabilities to buy infant formula [66]; therefore, it can facilitate accessing breastmilk substitutes and subsequently bottle-feeding practices which could lead to early breastfeeding cessation as found by Akter et al. (2010) [36].

In addition, in recent years, educated mothers in Bangladesh are more inclined to undergo cesarean section (C-section) delivery [67], which is 77.6% among women with secondary or higher education [68]. C-section delivery is one of the major risk factors for not initiating early breastfeeding immediately after birth [69–71]. C-section deliveries are conducted using anesthesia. Hence, it becomes very difficult for mothers to recover within one hour of birth and begin breastfeeding. In addition, maternal tiredness, respiratory distress among newborns, and post-surgical procedures may contribute to not initiating breastfeeding within one hour after delivery [39]. These might be the reasons why parental, particularly maternal, education is negatively associated with early initiation of breastfeeding.

In addition, working mothers have higher levels of income than unemployed mothers, and thereby greater access to better choices of food [47]. Researchers found that employed mothers have better knowledge on child health and nutrition, which could influence feeding practices positively [47, 72, 73], provide improved access to related information [72], and are better at seeking healthcare [38] than their unemployed counterparts. However, the greater wealth and agency of educated mothers also means that they have increased access to artificial breast milk substitutes and processed foods, which can be detrimental to their offspring [35, 46].

This review found that mothers with lower educational attainment had better breastfeeding practices than those with more education. One reason behind this phenomenon, as mentioned by some of the reviewed studies, is the difficulty of breastfeeding for the educated mothers engaging in full-time employment [6, 21]. Considering the perspective of women employed in the ready-made garments (RMG) sector could be a useful example in understanding why employment and educational attainment is not fully supportive of proper IYCF practices. This sector plays an important role in the economy of Bangladesh, employing more women than any other sector [74]. Among these, 45.3% of garment workers have at least primary education, 29.8% have an education level less than SSC (secondary school certificate), and 24.3% have a level of complete SSC (secondary) education [75]. Researchers showed that approximately 76% of mothers working in the RMG sector knew that the babies should be exclusively breastfed up to six months of their age, though only four out of ten (44%) were found to practice exclusive breastfeeding [76]. Translating the knowledge into practice is difficult for the mothers working in the RMG sector due to structural barriers at the workplace, and most mothers introduce formula feeding as early as two months after birth [19, 77, 78]. Maternity leave in Bangladesh is not strictly maintained and varies by types of employment, nursing breaks remain unofficial, and childcare facilities at workplaces are extremely scarce [19, 79]. Employed mothers also have increased household income and greater affordability and desirability for commercial breastmilk substitutes. Another potential cause is that employed mothers are more likely to introduce complementary food earlier, which leads to quicker termination of breastfeeding [80]. Meanwhile, uneducated mothers perceive breastfeeding as a cost-effective way to feed their babies compared to buying breast milk substitutes and other foods and thus have better breastfeeding practices compared to their counterparts [81].

In most of the studies, the researchers mainly explained how maternal education is associated with IYCF, whereas its association with paternal education was largely disregarded. However, educated women in Bangladesh tend to have educated husbands [82]. Therefore, even after considering the possible multicollinearity, the associations could be expected to be the same for paternal education. Like the employment of educated mothers, employment of educated fathers has influences on the child feeding practices. Employed fathers often cannot provide enough time in supporting mothers in child feeding [19] while it has been reported that mothers with supportive husbands are more likely to practice exclusive breastfeeding than their counterparts [83]. Our findings are consistent with the findings from other South Asian countries. Parental education was found to be negatively associated with optimal breastfeeding in studies conducted in India and Pakistan [81, 84–87]. The common reason behind this scenario is that educated mothers are more likely to be in employment than uneducated mothers; unsupportive working environments with no or limited breastfeeding opportunities could explain poor breastfeeding practices [88]. In contrast to the negative associations, other studies also found that parental education was positively associated with breastfeeding in South Asian countries [84, 87, 89–94]. According to these studies, the reason is probably because educated mothers are more likely to access healthcare messages, aware of healthy and timely child feeding practices, and more capable of making informed health related decisions [90, 91]. Furthermore, education plays a positive role in changing traditional beliefs, improving the attitudes of mothers, and perceiving the healthcare messages that catalyze the improved complementary feeding practices [39].

Proposed reasons for this relationship include socioeconomic differences in attitudes toward breastfeeding, improved health literacy and knowledge of breastfeeding benefits, higher self-efficacy, greater success in reaching educated women with breastfeeding-promoting messages, working in a job that allows continuing breastfeeding as well as overall greater social support for educated women [95–97]. However, it is difficult to argue that promoting maternal education is sufficient to improve IYCF practices when numerous structural and cultural barriers exist, including lack of funding for breastfeeding-promoting initiatives, violations of the International Code of Marketing of Breast-milk Substitutes, and misalignment of regional or country-level scientific opinions with the WHO global recommendations on exclusive breastfeeding [98–100].

In summary, parental education seems to be positively associated with complementary feeding practices; however, in most of the cases, the association is negative for breastfeeding in Bangladesh despite that breastfeeding could entail considerable health expenditure savings, minimize economic loss, and result in various socio-economic benefits in the long run [101–105]. The authors therefore put forward investment into IYCF promotion and protection as a priority in Bangladesh considering the associated health and economic benefits.

### Policy implications and recommendations

In Bangladesh, 19 policy documents in favor of IYCF promotion were identified [4]. However, substantial gaps in terms of putting policies into action, population coverage, inter-sectoral coordination, and engagement of the non-public sector were identified. The analysis also suggested a need for strategies to engage relevant stakeholders in implementation of these policies that support IYCF in Bangladesh. These suggestions are in consensus with our findings. For example, breastfeeding practices of employed mothers should be supported by policy implementation such as ensuring mandatory six-month maternity leave with full compensation, breastfeeding creches at workplaces, and adequate breastfeeding breaks. To promote breastfeeding and discourage breastmilk substitutes among educated mothers, innovative interventions using online platforms could be considered [106, 107].

On the other hand, complementary feeding practices were poor among mothers with lower educational attainment than their counterparts. Several steps could also be taken to improve this group's complementary feeding practices, including creating employment for uneducated mothers, strengthening existing nutrition interventions and services targeting uneducated and lower socio-economic mothers, increasing community outreach to reach a maximum number of underprivileged mothers, and demonstrating how to prepare proper complementary diet using locally available and affordable food items. Finally, the long-term solution considers reducing the socio-economic inequities so that mothers get access to resources for improving their IYCF practices through education and employment.

### Strengths and limitations

This is the first systematic review to observe the association between parental education and IYCF practices in Bangladesh. Additionally, it is the first of its kind in the South Asian context. In this review, most of the included studies considered nationally representative data (BDHS data), and a number of variables were adjusted for during analysis; therefore, the likelihood of estimated association is expected to provide a reliable and valid estimate regarding association between exposure and outcome.

Several limitations could also be mentioned. Only four databases were searched to collect the evidence. Meta-analysis was not performed; therefore, conclusions are based on findings from individual studies. Finally, the findings could be generalized in similar contexts but not in other settings, for example high-income countries.

## Conclusions

According to findings of the majority of the included studies, parental education is positively associated with complementary feeding practices but negatively associated with breastfeeding-related indicators. Therefore, the role of parental education in breastfeeding their infants and young children is ambiguous in the Bangladeshi context. Common reasons behind educated mothers not ensuring optimum breastfeeding include their engagement in employment and unsupportive environments for breastfeeding. Both parental education and standard IYCF practices are equally important for national development. Therefore, it is important to put policies into action so that educated mothers can ensure optimum breastfeeding and uneducated mothers get access to resources for ensuring recommended complementary feeding.

### Supplementary Information

**Additional file 1.**

## Data Availability

All the associated data is provided as supplementary material.
