# Publisher Correction: How is parental education associated with infant and young child feeding in Bangladesh? A systematic literature review

**DOI:** 10.1186/s12889-023-15823-4

**Published:** 2023-09-25

**Authors:** Plabon Sarkar, M. A. Rifat, Progati Bakshi, Imdadul Haque Talukdar, Sarah M. L. Pechtl, Tobias Lindström Battle, Sanjib Saha

**Affiliations:** 1 Caritas Bangladesh, 2, Outer Circular Road, Shantibagh, Dhaka, 1217 Bangladesh; 2 Department of Global Public Health, Karolinska Institutet, Stockholm, 17177 Sweden; 3 Department of Food and Agroprocess Engineering, Bangabandhu Sheikh Mujibur Rahman Science and Technology University, Imdadul Haque Talukdar, Gopalganj, 8100 Bangladesh; 4 Department of Learning, Informatics, Management and Ethics (LIME), Karolinska Institutet, Stockholm, Sweden; 5 Department of Clinical Sciences, Health Economics Unit, Lund University, Lund, 22381 Sweden

**BMC Public Health (2023) 23:510**

10.1186/s12889-023-15173-1

During the publication process 2 errors were introduced in the PDF publication:

1) In page 26, the text “Not introduction of solid, semi-solid or soft food (6-8 months)” was missing in the IYCF component column.

2) In page 27, the text “Tariqujjaman et al. (2022) [61]” was missing in the Author, year column.

The original article has been updated to correct these errors. The publisher apologizes to the authors & readers for the inconvenience.

