# Supplementary material for: How is parental education associated with infant and young child feeding in Bangladesh? a systematic literature review
Source: BMC Public Health. 2023 Mar 17;23:510. doi: 10.1186/s12889-023-15173-1 (PMC10022043; doi:10.1186/s12889-023-15173-1)
Supplement: Supplementary file 1 — Additional file 1. [file 12889_2023_15173_MOESM1_ESM.docx]

**Supplementary materials**

**Title:** How is parental education associated with infant and young child feeding in Bangladesh? A systematic literature review

**Index**

| **No.** | **Name of the item** | **Page no** |
| --- | --- | --- |
| 1 | Search strategy | 01-04 |
| 2 | Quality assessment of the included articles | 05-07 |

### **Search strategy**

**PubMed**

| #13 | #12 AND #11 AND #7 | 145 |
| --- | --- | --- |
| #12 | Search: Bangladesh[MeSH] OR Bangladesh OR Bangladeshi Sort by: Most Recent | [33,153](https://pubmed.ncbi.nlm.nih.gov/?term=Bangladesh%5BMeSH%5D+OR+Bangladesh+OR+Bangladeshi&sort=date) |
| #11 | #10 OR #9 OR #8 | [75,245](https://pubmed.ncbi.nlm.nih.gov/?term=%28%28%22parental+education%22+OR+%22parental+literacy%22+OR+%22parental+schooling%22+OR+%22caregiver%E2%80%99s+education%22+OR+%22caregiver%E2%80%99s+schooling%22+OR+%22caregivers%E2%80%99+education%22+OR+%22caregivers%E2%80%99+literacy%22+OR+%22educated+parents%22+OR+%22uneducated+parents%22%29+OR+%28%22paternal+education%22+OR+%22fathers%E2%80%99+education%22+OR+%22father%E2%80%99s+education%22+OR+%22paternal+schooling%22+OR+%22father%E2%80%99s+schooling%22+OR+%22paternal+literacy%22+OR+%22father%E2%80%99s+literacy%22+OR+%22educated+fathers%22%29%29+OR+%28%28maternal+educational+status%5BMeSH+Terms%5D%29+OR+%28status%2C+maternal+educational%5BMeSH+Terms%5D%29+OR+%22maternal+education%22+OR+%22mothers%E2%80%99+education%22+OR+%22mother%E2%80%99s+education%22+OR+%22maternal+schooling%22+OR+%22mothers%E2%80%99+schooling%22+OR+%22mother%E2%80%99s+schooling%22+OR+%22maternal+literacy%22+OR+%22mother%E2%80%99s+literacy%22+OR+%22mother%E2%80%99s+literacy%22+OR+%22educated+mothers%22+OR+%22uneducated+mothers%22%29&sort=date) |
| #10 | Search: "parental education" OR "parental literacy" OR "parental schooling" OR "caregiver’s education" OR "caregiver’s schooling" OR "caregivers’ education" OR "caregivers’ literacy" OR "educated parents" OR "uneducated parents" Sort by: Most Recent | [8,266](https://pubmed.ncbi.nlm.nih.gov/?term=%E2%80%9Cparental+education%E2%80%9D+OR+%E2%80%9Cparental+literacy%E2%80%9D+OR+%E2%80%9Cparental+schooling%E2%80%9D+OR+%E2%80%9Ccaregiver%E2%80%99s+education%E2%80%9D+OR+%E2%80%9Ccaregiver%E2%80%99s+schooling%E2%80%9D+OR+%E2%80%9Ccaregivers%E2%80%99+education%E2%80%9D+OR+%E2%80%9Ccaregivers%E2%80%99+literacy%E2%80%9D+OR+%22educated+parents%22+OR+%22uneducated+parents%22&sort=date) |
| #9 | Search: "paternal education" OR "fathers’ education" OR "father’s education" OR "paternal schooling" OR "father’s schooling" OR "paternal literacy" OR "father’s literacy" OR "educated fathers" Sort by: Most Recent | [1,600](https://pubmed.ncbi.nlm.nih.gov/?term=%E2%80%9Cpaternal+education%E2%80%9D+OR+%E2%80%9Cfathers%E2%80%99+education%E2%80%9D+OR+%E2%80%9Cfather%E2%80%99s+education%E2%80%9D+OR+%E2%80%9Cpaternal+schooling%E2%80%9D+OR+%E2%80%9Cfather%E2%80%99s+schooling%E2%80%9D+OR+%E2%80%9Cpaternal+literacy%E2%80%9D+OR+%E2%80%9Cfather%E2%80%99s+literacy%E2%80%9D+OR+%E2%80%9Ceducated+fathers%E2%80%9D&sort=date) |
| #8 | Search: (maternal educational status[MeSH Terms]) OR (status, maternal educational[MeSH Terms]) OR "maternal education" OR "mothers’ education" OR "mother’s education" OR "maternal schooling" OR "mothers’ schooling" OR "mother’s schooling" OR "maternal literacy" OR "mother’s literacy" OR "mother’s literacy" OR "educated mothers" OR "uneducated mothers" Sort by: Most Recent | [67,427](https://pubmed.ncbi.nlm.nih.gov/?term=%28maternal+educational+status%5BMeSH+Terms%5D%29+OR+%28status%2C+maternal+educational%5BMeSH+Terms%5D%29+OR+%E2%80%9Cmaternal+education%E2%80%9D+OR+%E2%80%9Cmothers%E2%80%99+education%E2%80%9D+OR+%E2%80%9Cmother%E2%80%99s+education%E2%80%9D+OR+%E2%80%9Cmaternal+schooling%E2%80%9D+OR+%E2%80%9Cmothers%E2%80%99+schooling%E2%80%9D+OR+%E2%80%9Cmother%E2%80%99s+schooling%E2%80%9D+OR+%E2%80%9Cmaternal+literacy%E2%80%9D+OR+%E2%80%9Cmother%E2%80%99s+literacy%E2%80%9D+OR+%E2%80%9Cmother%E2%80%99s+literacy%E2%80%9D+OR+%E2%80%9Ceducated+mothers%E2%80%9D+OR+%E2%80%9Cuneducated+mothers%E2%80%9D&sort=date) |
| #7 | #6 OR #5 OR #4 OR #3 OR #2 OR #1 | [1,117,832](https://pubmed.ncbi.nlm.nih.gov/?term=%28%28%28%28%28%22Minimum+meal+frequency%22+OR+%22Minimum+acceptable+diet%22+OR+%22Dietary+diversity%22%29+OR+%28complementary+feeding%5BMeSH+Terms%5D+OR+complementary+feedings%5BMeSH+Terms%5D+OR+%22Complementary+feeding%22+OR+complement%2A+OR+supplement%2A+OR+%22food+complement%22+OR+%22Appropriate+complementary+feeding%22%29%29+OR+%28%22infant+feeding%22+OR+%22neonatal+feeding%22+OR+%22child+feeding%22+OR+%22baby+feeding%22+OR+%22Infant+and+young+child+feeding%22+OR+infant+food%5BMeSH+Terms%5D+OR+infant+foods%5BMeSH+Terms%5D+OR+IYCF%29%29+OR+%28weaning+OR+%22weaning+practice%22+OR+weaning%5BMeSH+Terms%5D+OR+%22weaning+food%22+OR+%22weaning+foods%22%29%29+OR+%28%22Prelacteal+feeding%22+OR+%22Bottle+feeding%22+OR+bottle+feeding%5BMeSH+Terms%5D+OR+bottle+feedings%5BMeSH+Terms%5D+OR+%22formula+feeding%22+OR+%22breastmilk+substitute%22+OR+infant+formula%5BMeSH+Terms%5D+OR+infant+formulas%5BMeSH+Terms%5D%29%29+OR+%28Breastfeeding%5BMeSH%5D+OR+Breastfeeding+OR+%22Early+Breastfeeding%22+OR+Colostrum%5BMeSH%5D+OR+Colostrum+OR+%22Colostrum+feeding%22+OR+%22Early+initiation+of+breastfeeding%22+OR+%22Exclusive+breastfeeding%22+OR+%22Timely+breastfeeding%22+OR+%28breastmilk+expression%5BMeSH+Terms%5D%29+OR+%28breastmilk+expressions%5BMeSH+Terms%5D%29+OR+%22Breastfeeding+continuation%22+OR+%22Continuation+of+breastfeeding%22+OR+%22Age+appropriate+breastfeeding%22+OR+feeding+breast+OR+lactation+OR+lactation%5BMeSH+Terms%5D+OR+breastfeed+OR+breast+fed%29&sort=date) |
| #6 | Search: "Minimum meal frequency" OR "Minimum acceptable diet" OR "Dietary diversity" Sort by: Most Recent | [1,995](https://pubmed.ncbi.nlm.nih.gov/?term=%22Minimum+meal+frequency%22+OR+%22Minimum+acceptable+diet%22+OR+%22Dietary+diversity%22&sort=date) |
| #5 | Search: complementary feeding[MeSH Terms] OR complementary feedings[MeSH Terms] OR "Complementary feeding" OR complement* OR supplement* OR "food complement" OR "Appropriate complementary feeding" Sort by: Most Recent | [995,733](https://pubmed.ncbi.nlm.nih.gov/?term=complementary+feeding%5BMeSH+Terms%5D+OR+complementary+feedings%5BMeSH+Terms%5D+OR+%22Complementary+feeding%22+OR+complement%2A+OR+supplement%2A+OR+%E2%80%9Cfood+complement%E2%80%9D+OR+%22Appropriate+complementary+feeding%22&sort=date) |
| #4 | Search: "infant feeding" OR "neonatal feeding" OR "child feeding" OR "baby feeding" OR "Infant and young child feeding" OR infant food[MeSH Terms] OR infant foods[MeSH Terms] OR IYCF Sort by: Most Recent | [22,103](https://pubmed.ncbi.nlm.nih.gov/?term=%22infant+feeding%22+OR+%22neonatal+feeding%22+OR+%22child+feeding%22+OR+%22baby+feeding%22+OR+%22Infant+and+young+child+feeding%22+OR+infant+food%5BMeSH+Terms%5D+OR+infant+foods%5BMeSH+Terms%5D+OR+IYCF&sort=date) |
| #3 | Search: weaning OR "weaning practice" OR weaning[MeSH Terms] OR "weaning food" OR "weaning foods" Sort by: Most Recent | [51,586](https://pubmed.ncbi.nlm.nih.gov/?term=weaning+OR+%22weaning+practice%22+OR+weaning%5BMeSH+Terms%5D+OR+%22weaning+food%22+OR+%22weaning+foods%22&sort=date) |
| #2 | Search: "Prelacteal feeding" OR "Bottle feeding" OR bottle feeding[MeSH Terms] OR bottle feedings[MeSH Terms] OR "formula feeding" OR "breastmilk substitute" OR infant formula[MeSH Terms] OR infant formulas[MeSH Terms] Sort by: Most Recent | [11,392](https://pubmed.ncbi.nlm.nih.gov/?term=%22Prelacteal+feeding%22+OR+%22Bottle+feeding%22+OR+bottle+feeding%5BMeSH+Terms%5D+OR+bottle+feedings%5BMeSH+Terms%5D+OR+%E2%80%9Cformula+feeding%E2%80%9D+OR+%E2%80%9Cbreastmilk+substitute%E2%80%9D+OR+infant+formula%5BMeSH+Terms%5D+OR+infant+formulas%5BMeSH+Terms%5D&sort=date) |
| #1 | Search: Breastfeeding[MeSH] OR Breastfeeding OR "Early Breastfeeding" OR Colostrum[MeSH] OR Colostrum OR "Colostrum feeding" OR "Early initiation of breastfeeding" OR "Exclusive breastfeeding" OR "Timely breastfeeding" OR (breastmilk expression[MeSH Terms]) OR (breastmilk expressions[MeSH Terms]) OR "Breastfeeding continuation" OR "Continuation of breastfeeding" OR "Age appropriate breastfeeding" OR feeding breast OR lactation OR lactation[MeSH Terms] OR breastfeed OR breast fed Sort by: Most Recent | [139,245](https://pubmed.ncbi.nlm.nih.gov/?term=Breastfeeding%5BMeSH%5D+OR+Breastfeeding+OR+%22Early+Breastfeeding%22+OR+Colostrum%5BMeSH%5D+OR+Colostrum+OR+%22Colostrum+feeding%22+OR+%22Early+initiation+of+breastfeeding%22+OR+%22Exclusive+breastfeeding%22+OR+%22Timely+breastfeeding%22+OR+%28breastmilk+expression%5BMeSH+Terms%5D%29+OR+%28breastmilk+expressions%5BMeSH+Terms%5D%29+OR+%22Breastfeeding+continuation%22+OR+%22Continuation+of+breastfeeding%22+OR+%22Age+appropriate+breastfeeding%22+OR+feeding+breast+OR+lactation+OR+lactation%5BMeSH+Terms%5D+OR+breastfeed+OR+breast+fed&sort=date) |

**Web of Science**

| #13 | #12 AND #11 AND #7 | [116](https://www-webofscience-com.proxy.kib.ki.se/wos/woscc/summary/3637c2fd-1893-4fc4-b3ff-17cbbbd67722-47f82302/relevance/1) |
| --- | --- | --- |
| #12 | ALL=(Bangladesh OR Bangladeshi) | [79,866](https://www-webofscience-com.proxy.kib.ki.se/wos/woscc/summary/9c5037e9-53b4-4e11-bcc4-a4a6148e3b29-47f819ec/relevance/1) |
| #11 | #10 OR #9 OR #8 | [15,869](https://www-webofscience-com.proxy.kib.ki.se/wos/woscc/summary/0de19e19-1835-4574-b5d0-6ce69e9ba12e-47f812a6/relevance/1) |
| #10 | ALL=(“parental education” OR “parental literacy” OR “parental schooling” OR “caregiver’s education” OR “caregiver’s schooling” OR “caregivers’ education” OR “caregivers’ literacy” OR "educated parents" OR "uneducated parents") | [6,512](https://www-webofscience-com.proxy.kib.ki.se/wos/woscc/summary/7951c9fc-3fde-4de3-ba0e-0e89952e82f6-47f806bf/relevance/1) |
| #9 | ALL=(“paternal education” OR “fathers’ education” OR “father’s education” OR “paternal schooling” OR “father’s schooling” OR “paternal literacy” OR “father’s literacy” OR “educated fathers”) | [1,445](https://www-webofscience-com.proxy.kib.ki.se/wos/woscc/summary/29bd6cf2-8f0b-4c6f-a67b-19b4cd21e0e5-47f800fb/relevance/1) |
| #8 | ALL=(“maternal education” OR “mothers’ education” OR “mother’s education” OR “maternal schooling” OR “mothers’ schooling” OR “mother’s schooling” OR “maternal literacy” OR “mother’s literacy” OR “mother’s literacy” OR “educated mothers” OR “uneducated mothers”) | [8,869](https://www-webofscience-com.proxy.kib.ki.se/wos/woscc/summary/89215ac5-5bd0-4506-bc0e-c6b2e3372450-47f7fa79/relevance/1) |
| #7 | #6 OR #5 OR #4 OR #3 OR #2 OR #1 | [1,555,351](https://www-webofscience-com.proxy.kib.ki.se/wos/woscc/summary/6ae7bcd9-07c0-4537-9a41-75f6f801bbcf-47f7e6bc/relevance/1) |
| #6 | ALL=("minimum meal frequency" OR "minimum acceptable diet" OR "dietary diversity") | [3,129](https://www-webofscience-com.proxy.kib.ki.se/wos/woscc/summary/50638bde-8870-49dc-816f-d6bc99785bf7-47f7e14e/relevance/1) |
| #5 | ALL=("complementary feeding" OR complement* OR supplement* OR “food complement” OR "appropriate complementary feeding") | [1,415,687](https://www-webofscience-com.proxy.kib.ki.se/wos/woscc/summary/d063bb80-3b10-4176-889b-eae0b906acc7-47f7daef/relevance/1) |
| #4 | ALL=("infant feeding" OR "neonatal feeding" OR "child feeding" OR "baby feeding" OR "infant and young child feeding" OR IYCF) | [8,697](https://www-webofscience-com.proxy.kib.ki.se/wos/woscc/summary/0d856026-9099-4eb5-9353-ca6dcc0612e0-47f7d52c/relevance/1) |
| #3 | ALL=(weaning OR "weaning practice" OR "weaning food" OR "weaning foods") | [57,690](https://www-webofscience-com.proxy.kib.ki.se/wos/woscc/summary/66c62766-77e3-4c13-b955-0e833f6c730d-47f7ceb4/relevance/1) |
| #2 | ALL=("prelacteal feeding" OR "bottle feeding" OR “formula feeding” OR “infant formula” OR “breastmilk substitute”) | [9,580](https://www-webofscience-com.proxy.kib.ki.se/wos/woscc/summary/89f536e5-43b8-4b80-ba38-351a088ba244-47f7c561/relevance/1) |
| #1 | ALL=(breastfeeding OR "early breastfeeding" OR colostrum OR "colostrum feeding" OR "early initiation of breastfeeding" OR "exclusive breastfeeding" OR "timely breastfeeding" OR "breastfeeding continuation" OR "continuation of breastfeeding" OR "age appropriate breastfeeding" OR “feeding breast” OR lactation OR breastfeed OR “breast fed”) | [100,855](https://www-webofscience-com.proxy.kib.ki.se/wos/woscc/summary/f6dda8cd-b487-461d-b21e-43d47dd05a26-47f7bb60/relevance/1) |

**Embase**

| #13 | #7 AND #11 AND #12 | [111](https://www.embase.com/) |
| --- | --- | --- |
| #12 | bangladesh OR bangladeshi | [47,354](https://www.embase.com/) |
| #11 | #8 OR #9 OR #10 | [14,349](https://www.embase.com/) |
| #10 | 'parental education' OR 'parental literacy' OR 'parental schooling' OR 'educated parents' OR 'uneducated parents' | [6,328](https://www.embase.com/) |
| #9 | 'paternal education' OR 'paternal schooling' OR 'paternal literacy' OR 'educated fathers' OR 'uneducated father' | [836](https://www.embase.com/) |
| #8 | 'maternal education' OR 'maternal schooling' OR 'maternal literacy' OR 'educated mothers' OR 'uneducated mothers' | 7,820 |
| #7 | #1 OR #2 OR #3 OR #4 OR #5 OR #6 | 3,170,184 |
| #6 | 'minimum meal frequency' OR 'minimum acceptable diet' OR 'dietary diversity' | [2,344](https://www.embase.com/) |
| #5 | 'complementary feeding' OR complement* OR supplement* OR 'food complement' OR 'appropriate complementary feeding' | [2,991,107](https://www.embase.com/) |
| #4 | 'infant feeding' OR 'neonatal feeding' OR 'child feeding' OR 'baby feeding' OR 'infant and young child feeding' OR iycf | [16,115](https://www.embase.com/) |
| #3 | weaning OR 'weaning practice' OR 'weaning food' OR 'weaning foods' | [57,139](https://www.embase.com/) |
| #2 | 'prelacteal feeding' OR 'bottle feeding' OR 'formula feeding' OR 'infant formula' OR 'breastmilk substitute' | [111](https://www.embase.com/) |
| #1 | 'breastfeeding'/exp OR breastfeeding OR 'early breastfeeding' OR 'colostrum'/exp OR colostrum OR 'colostrum feeding' OR 'early initiation of breastfeeding' OR 'exclusive breastfeeding'/exp OR 'exclusive breastfeeding' OR 'timely breastfeeding' OR 'breastfeeding continuation' OR 'continuation of breastfeeding' OR 'age appropriate breastfeeding' OR 'feeding breast'/exp OR 'feeding breast' OR 'lactation'/exp OR lactation OR breastfeed OR 'breast fed' | [151,978](https://www.embase.com/) |

**Keywords used to conduct manual searching in Google Scholar:**

Infant feeding, child feeding, breastfeeding, complementary feeding, exclusive breastfeeding, bottle feeding, and Bangladesh

### **Quality assessment of the included articles**

Table: Quality of the included study according to Newcastle-Ottawa assessment scale

| Cross-sectional | | | | | | | | | |
| --- | --- | --- | --- | --- | --- | --- | --- | --- | --- |
| Author, year | Selection | | | | Comparability | Outcome | | Overall quality | |
|  | Representativeness of the sample | Sample size | Non-respondent | Exposure assignment |  | Outcome assessment | Statistical accuracy |  |  |
| Ahmed, 1999 | a* | a* | a* | a** | b | c* | a* | 07 | |
| Ahmmed 2021 | a* | a* | a* | a** | a** | a** | a* | 10 | |
| Akter, 2010a | a* | a* | a* | b* | a** | a** | a* | 09 | |
| Akter, 2010b | a* | a* | a* | b* | a** | a** | a* | 09 | |
| Akter 2016 | a* | a* | a* | b* | b | a** | a* | 07 | |
| Ali, 2019 | a* | a* | a* | b* | a** | a** | a* | 09 | |
| Al Mamun, 2022 | a* | a* | a* | b* | a** | b** | a* | 09 | |
| Basnet 2020 | a* | a* | a* | b* | a** | a** | a* | 09 | |
| Blackstone, 2018 | a* | a* | a* | b* | a** | a** | a* | 09 | |
| Chowdhury, 2016 | a* | a* | a* | b* | a** | a** | a* | 09 | |
| Dintyala, 2020 | a* | a* | a* | b* | a** | a** | a* | 09 | |
| Giashuddin, 2004 | a* | a* | a* | b* | a** | a** | a* | 09 | |
| Hasan 2020 | a* | a* | a* | b* | a** | a** | a* | 09 | |
| Hossain, 2018 | a* | a* | a* | b* | a** | a** | a* | 09 | |
| Islam, 2019 | a* | a* | a* | b* | a** | a** | a* | 09 | |
| Islam, 2019 | a* | a* | a* | b* | a** | a** | a* | 09 | |
| Jain, 1981 | a* | a* | a* | b* | a** | a** | a* | 09 | |
| Kabir, 2012 | a* | a* | a* | b* | a** | a** | a* | 09 | |
| Karim, 2019 | a* | a* | a* | b* | a** | a** | a* | 09 | |
| Khan, 2020 | a* | a* | a* | b* | a** | a** | a* | 08 | |
| Khan, 2022 | a* | a* | a* | b* | a** | a** | a* | 09 | |
| Mihrshahi 2010 | a* | a* | a* | b* | a** | a** | a* | 09 | |
| Na, 2018 | a* | a* | a* | b* | a** | a** | a* | 09 | |
| Nguyen, 2013 | a* | a* | a* | b* | a** | a** | a* | 09 | |
| Rahman, 2011 | a* | a* | a* | b* | a** | a** | a* | 09 | |
| Rana, 2020 | a* | a* | a* | b* | a** | b** | a* | 09 | |
| Sakib, 2021 | a* | a* | a* | b* | b | a** | a* | 08 | |
| Sen, 2020 | a* | a* | a* | b* | a** | a** | a* | 09 | |
| Senarath, 2012 | a* | a* | a* | b* | a** | a** | a* | 09 | |
| Shahjahan, 2012 | a* | a* | a* | b* | a** | a** | a* | 09 | |
| Sundaram, 2013 | a* | a* | a* | b* | a** | a** | a* | 09 | |
| Tariqujjaman, 2022 | a* | a* | a* | b* | a** | a** | a* | 09 | |
| Randomized controlled trial | | | | | | | | | |
| Author, year | Selection | | | | Comparability | Exposure | | | Overall Quality |
|  | Case definition | Representativeness of case | Selection of control | Definition of control |  | Ascertainment | Same methods | Non-response rate |  |
| Campbell, 2016 | a* | a* | a* | a* | a*b* | e | a* | a* | 08 |
| Cohort or longitudinal^∝^ | | | | | | | | | |
| Author, year | Selection | | | | Comparability | Outcome | | | Overall Quality |
|  | Representativeness of exposed cohort (ISA) | Selection of non-exposed cohort (GSA) | Ascertainment of exposure | Absence of outcome interest at the beginning |  | Assessment of outcome | Adequate follow-up | Adequacy of follow-up |  |
| Rahman, 2020 | a* | a* | a* | Not applicable | a*b* | b* |  | d | 06 |

^∝^The questions were contextualized from NOS
